# Eco-friendly tassel-derived activated carbon for efficient dye removal in wastewater treatment

**DOI:** 10.1038/s41598-025-28825-6

**Published:** 2025-12-02

**Authors:** Mona Moheb, Ahmad M. El-Wakil, Saadia M. Waly, Fathi S. Awad

**Affiliations:** Chemistry Department, Faculty of Science, Mansoura University, Mansoura, 35516 Egypt

**Keywords:** Environmental remediation, Sustainable adsorbents, Hazardous dyes, Tassel-Activated carbon, Chemistry, Engineering, Environmental sciences, Materials science

## Abstract

This study introduces a sustainable method for wastewater treatment by creating activated carbon from the fast-growing tassels of *Phragmites australis*, an invasive and low-cost biomass source. Its novelty lies in being the first to use the tassel part (inflorescence) of *Phragmites australis* as a precursor for activated carbon, demonstrating its excellent ability to remove Alizarin Red S (ARS), an anionic dye not previously studied with any *Phragmites australis*–based adsorbent. The resulting tassel-activated carbon (TAC) showed a high surface area (1166.16 m²/g), pore volume (1.5038 cm³/g), and abundant oxygen- and nitrogen-containing functional groups, confirmed by FTIR and XPS analyses. These structural features greatly improved its adsorption capacity for hazardous dyes like ARS and Methylene Blue (MB). The maximum adsorption capacities reached 541 mg/g for ARS and 860 mg/g for MB. Kinetic studies followed the pseudo-second-order model, indicating chemisorption, while equilibrium data fit the Langmuir isotherm, indicating monolayer adsorption. Thermodynamic results showed spontaneous and endothermic adsorption for ARS and spontaneous and exothermic adsorption for MB. TAC maintained high stability and reusability, retaining over 85% efficiency after seven adsorption–desorption cycles. Additionally, TAC effectively removed dyes from real water samples, including tap, Nile, and sewage water, demonstrating its practical potential. These findings position TAC as a promising, scalable, and eco-friendly adsorbent for industrial wastewater treatment, supporting sustainable waste valorization and cleaner aquatic environments.

**Supplementary Information:**

The online version contains supplementary material available at 10.1038/s41598-025-28825-6.

## Introduction

Global accelerated industrialization is contaminating freshwater with various chemical and biological pollutants. Effective wastewater treatment and recycling are crucial for obtaining freshwater, a precious resource. Industrial wastewater often contains harmful toxins, including dyes, antibiotics, and heavy metals^1,2^. Many dyes are poisonous and potentially carcinogenic, exhibiting resistance to degradation and contributing significantly to water pollution^3^. These dyes possess unique chromophore groups responsible for their color. Among them, dyes containing benzidine, azo, and anthraquinone moieties demand particular attention^4^. Methylene blue, a widely used cationic dye in the textile industry, is resistant to degradation, and its presence in aquatic environments hinders photosynthesis by obstructing light penetration. MB is also linked to serious health risks, including ocular, pulmonary, gastrointestinal, and mental health disorders^5–7^. Similarly, Alizarin Red S, an anthraquinone dye extensively used in textiles, presents significant challenges due to its fused aromatic structure, which resists degradation and threatens aquatic ecosystems and public health^8^. In environmental contexts, dye concentrations vary depending on the source of discharge. For example, methylene blue has been reported in textile effluents and industrial wastewater at levels ranging from a few mg/L up to 50 mg/L^6^, while alizarin red S is typically found in dyeing and printing wastewater at concentrations between 5 and 40 mg/L^9^. Although these values are lower than the high concentrations often used in laboratory batch studies, they highlight the persistence and ecological risk of such dyes. Accordingly, in this work we tested a wide concentration range (50–900 mg/L) to simulate both environmentally relevant levels and worst-case industrial scenarios, thereby ensuring that the adsorption performance of TAC is rigorously evaluated. The effective removal of these organic dyes from wastewater before discharge is essential to protect marine habitats. Researchers have explored various techniques for dye removal, including adsorption, flocculation, biodegradation, membrane filtering, and photocatalysis^10–12^. Among these, adsorption stands out due to its simplicity, low cost, and suitability for treating low-concentration pollutants^13^. However, commonly used adsorbents such as activated carbon, zeolites^14^, and polymeric materials often face limitations in selectively removing specific dyes or meeting sustainability criteria. The challenge lies in sourcing renewable, cost-effective, and environmentally friendly materials for activated carbon production^15^.

The common reed (*Phragmites australis*), a highly invasive wetland plant, presents a dual opportunity for environmental management. While it disrupts native biodiversity and obstructs waterways, it also offers benefits such as biomass production, renewable energy generation, and wastewater treatment^16,17^. Utilizing tassels of *Phragmites australis* (TPA) for activated carbon production not only supports photoremediation but also provides a sustainable feedstock for high-performance adsorbents. This approach addresses two key challenges: remediating polluted environments and producing cost-effective, reusable adsorbents for dye removal^18^.

Despite the extensive use of activated carbons for wastewater treatment, there is a notable research gap in exploring sustainable^19^, plant-based adsorbents specifically designed for removing dyes like ARS and MB. Existing studies lack adsorbents that combine high adsorption efficiency, sustainability, and reusability for these dyes. In this context, the present study not only introduces tassel-activated carbon (TAC) as a novel adsorbent but also addresses critical challenges associated with conventional adsorbents. These challenges include the lack of sustainable and low-cost precursors, limited reusability, and poor stability under practical conditions. TAC valorizes an abundant invasive biomass (*Phragmites australis* tassels) and combines high surface area, hierarchical porosity, and abundant functional groups with excellent regeneration ability, thereby offering a holistic solution that extends beyond laboratory adsorption performance. Although several adsorbents with higher maximum adsorption capacities have been reported^20–22^, many of them are derived from costly or non-renewable precursors, require complex synthesis steps, or lack regeneration potential. The novelty of TAC lies not only in its considerable adsorption efficiency (541 mg/g for ARS and 860 mg/g for MB) but also in its sustainability, facile synthesis, cost-effectiveness, and excellent reusability. These features highlight its practical applicability in real wastewater treatment and its added value to existing literature.

This study aims to fill this gap by developing a novel adsorbent from TPA, activated with phosphoric acid. The resulting tassel-activated carbon demonstrates high efficiency in removing ARS and MB, as evidenced by its superior adsorption capacity and stability. Characterization techniques such as FTIR, SEM, BET, and XPS were employed, and key adsorption parameters, including pH, temperature, and dye concentration, were systematically evaluated. TAC’s reusability and practicality highlight its potential as a sustainable solution for wastewater treatment.

## Experimental section

### Materials

Tassels of *Phragmites australis* (TPA) were sourced from Mansoura city as a carbon precursor. Methylene blue (C_16_H_18_ClN_3_S) and alizarin red S (C_14_H_7_NaO_7_S) were acquired from Sigma Chemical Co. without additional purification. The initial pH was performed using NaOH or HCl solutions and phosphoric acid, a chemical activation agent.

### Synthesis of tassel-activated carbon (TAC)

**Fig. 1:**
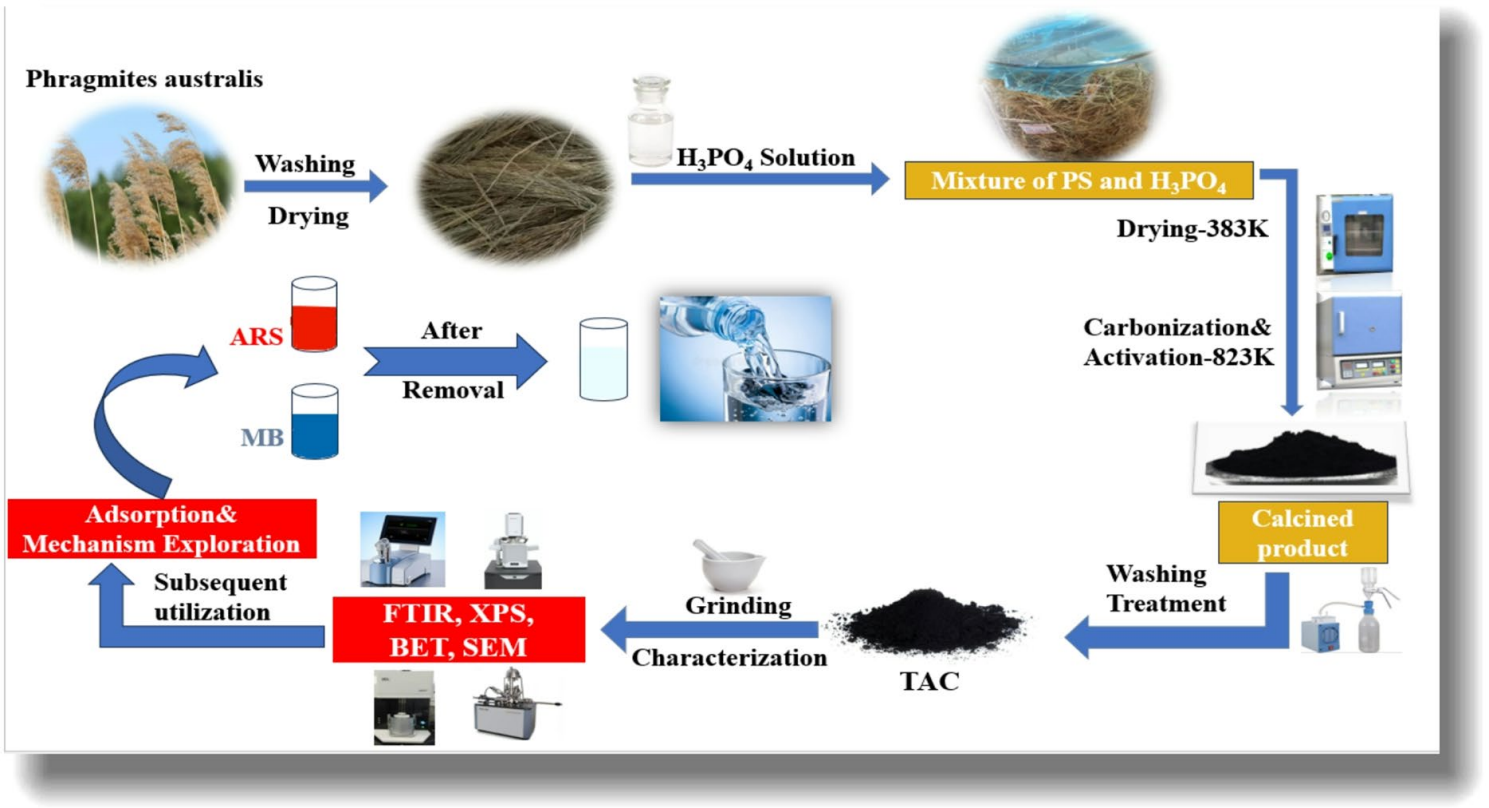
The general procedure for the preparation of tassel-activated carbon.

*Phragmites australis* (TPA) tassels were thoroughly washed with distilled water to remove dust and soluble impurities. The cleaned samples were dried at 110 °C for 12 h and then crushed. The resulting material, with a particle size range of 0.45 to 1.0 mm, was used as the precursor for activated carbon production. A total of 30 g of TPA was immersed in a solution of phosphoric acid (H₃PO₄, 50.0 ± 0.5% v/v) at an acid-to-solid mass ratio of 2:1. After impregnation at room temperature for 72 h, the mixture was dehydrated in an oven at 110 °C for 48 h to remove moisture. The samples were then subjected to pyrolysis in a muffle furnace at 550 °C for 3 h, with a heating rate of 5 °C/min under an inert nitrogen atmosphere. Once the materials reached room temperature, they were rinsed thoroughly with distilled water until the pH of the effluent stabilized. Finally, the activated carbon was dried at 105 °C for 10 h and sieved through a 120-mesh sieve. Figure 1 illustrates the overall synthesis of TAC-activated carbon.

### Characterization

The synthesized materials were characterized in terms of shape, surface chemical composition, crystal phase, functional groups, and textural properties. The textural parameters, including specific surface area, total pore volume, and pore size distribution, were determined using the Brunauer-Emmett-Teller (BET) nitrogen adsorption method at 77.35 K, with measurements taken on a Quantachrome/NOVA apparatus. The morphology and elemental composition of the activated carbon were analyzed using Scanning Electron Microscopy (SEM) with a Jeol JSM 6510LV instrument. Fourier Transform Infrared (FTIR) spectroscopy was employed to identify the surface functional groups, with spectra recorded from 4000 to 400 cm^–1^ using a Thermo Nicolet iS10 FT-IR spectrometer. Additionally, X-ray Photoelectron Spectroscopy (XPS) was utilized to examine the surface chemical states and elemental composition of the activated carbon, particularly before and during dye adsorption. The point of zero charge (pHpzc) was measured to evaluate the surface charge of the activated carbon particles.

### Adsorption investigations

Batch adsorption experiments were performed at ambient temperature (25 °C) by adding 0.005 g of TAC to 10 mL of dye solution at the desired initial concentration and optimal pH, followed by shaking at 350 rpm for 240 min. The effect of dye concentration (50–900 ppm) was studied at a constant adsorbent dosage (0.5 g/L) and agitation duration (240 min). The impact of adsorbent dosage (0.5–2 g/L) was assessed using fixed initial dye concentrations of 500 ppm for ARS and 800 ppm for MB, with an agitation duration of 90 min.

The influence of pH (2, 3, 4, 5, 6, 8, 9, and 10) on dye removal was investigated at fixed dye concentrations (500 ppm for ARS and 300 ppm for MB), an adsorbent dosage of 0.5 g/L, and agitation for 90 min. The pH was adjusted using 0.1 M NaOH and 0.1 M HCl. After reaching equilibrium, the adsorbent was separated, and the residual dye concentration was determined spectrophotometrically at the maximum wavelengths of 424.0 nm for ARS and 664.0 nm for MB.)calibration curves for both dyes are provided in the The final concentration (C_e_) was measured, and the percentage of dye removal (E%) and the adsorption capacity (q_e_) were calculated using the following Eqs. (1, 2)^23^:

1 $$\:\text{R}\text{e}\text{m}\text{o}\text{v}\text{a}\text{l}\:\text{\%}\:\:=\frac{100({C}_{0\:}-{C}_{e})}{{C}_{e}}$$

2 $$\:{q}_{e}=\left({C}_{0}-{C}_{e}\right)\times\:\frac{V}{W}$$

C_0_ denotes the initial dye concentration in the solution (mg/L), $\:{\text{C}}_{\text{e}}$ represents the equilibrium concentration (mg/L), q_e_ indicates the adsorption capacity (mg/g), V signifies the solution volume (L), and W refers to the adsorbent mass (g).

## Results and discussion

### Characterization

#### Textural and surface characterization of TAC adsorbent

**Fig. 2:**
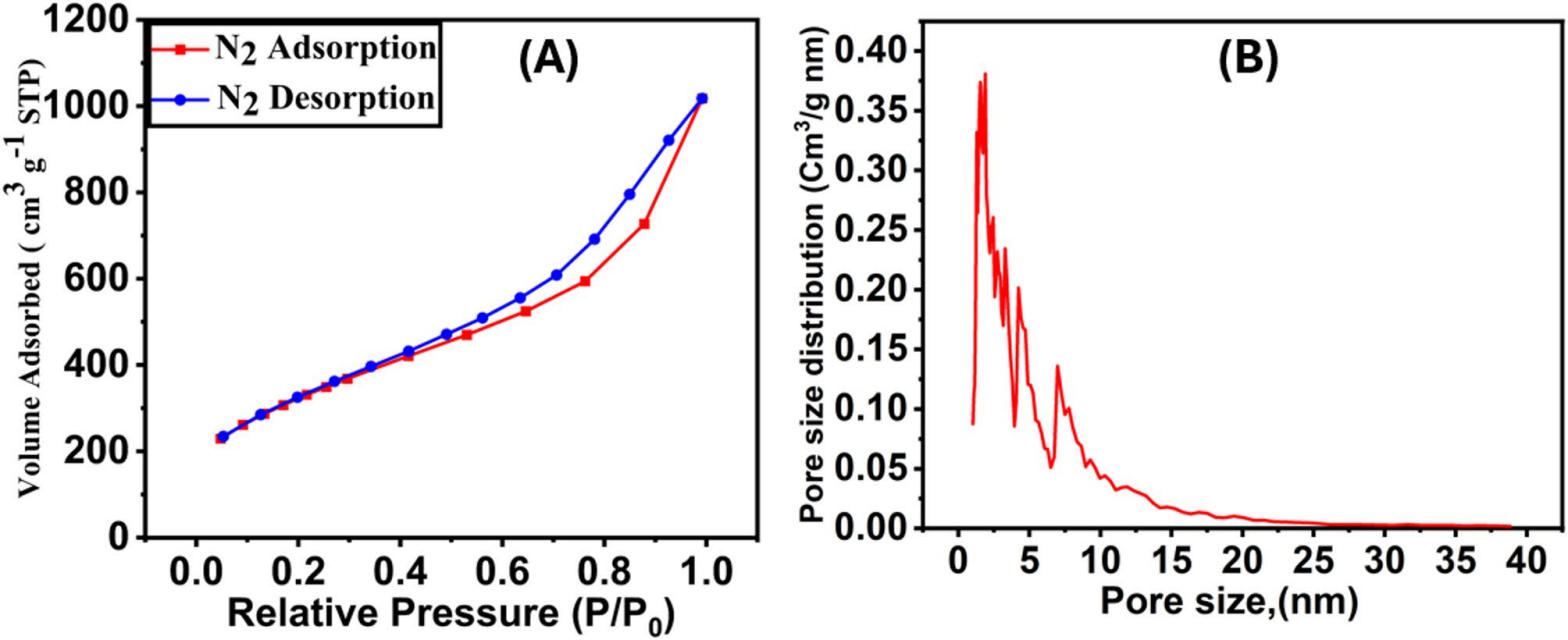
(A) The liquid nitrogen adsorption-desorption isotherms, (B) Pore size distribution.

The specific surface area and pore size distribution are critical physical parameters influencing dye adsorption on activated carbon. The textural properties of TAC are summarized in Table S1. The nitrogen (N_2_) adsorption/desorption isotherms of TAC (Fig. 2) are classified as type II according to IUPAC guidelines^24^, indicating the presence of macroporous structures. These isotherms display an H4-type hysteresis loop at relative pressures (p/p_0_) greater than 0.4, further supporting the existence of macroporosity. The pore size distribution (Fig. 2) confirms a hierarchical porous structure comprising micropores (< 2 nm) and mesopores (2–50 nm)^25^. The specific surface area and pore volume of TAC are measured to be 1166.16 m²/g and 1.5038 cm³/g, respectively, reflecting the material’s well-developed porous architecture. Such properties make TAC highly effective for the adsorption of methylene blue (MB) and alizarin red S (ARS).

#### FTIR analysis of functional groups on TAC adsorbent

**Fig. 3:**
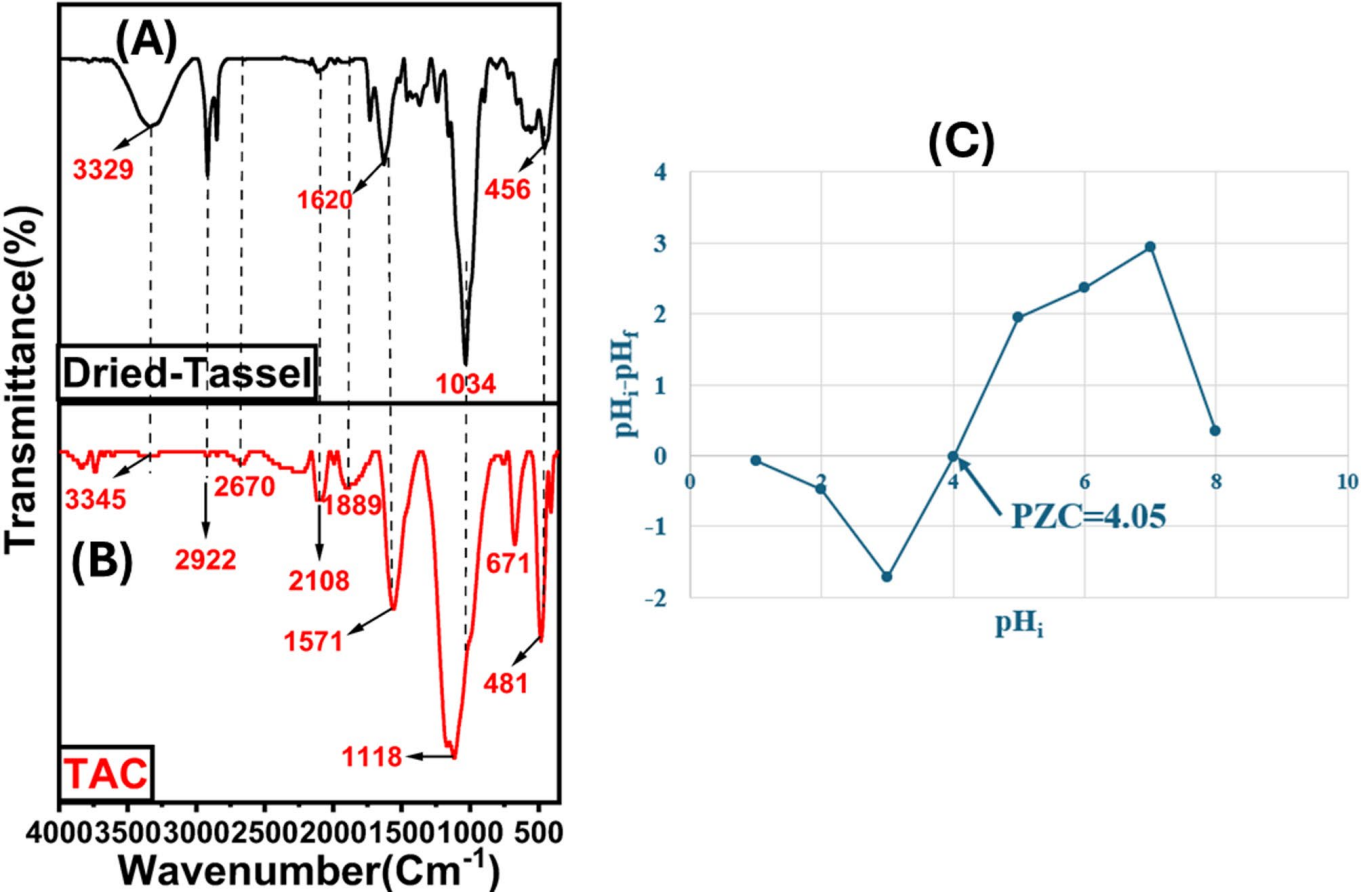
FTIR spectra of dried Tassel (A), and TAC (B) adsorbents. (C) Determination of pHpzc for TAC.

The FTIR spectra, presented in Fig. 3, reveal the functional groups present on the surface of the produced TAC adsorbent. The analysis highlights oxygen-containing functional groups on the sample surfaces. In the dried tassel, a prominent band at 3329 cm^–1^ corresponds to O–H and N–H stretching vibrations. These N–H groups mainly originate from proteins, amino sugars, and other nitrogenous compounds naturally present in the biomass associated with primary plant cell constituents such as lignin, cellulose, pectin, and hemicellulose^26^, due to their stretching vibrations. After activation, the intensity of this band decreases, indicating significant hydrogen removal from the plant material.The bands at 2922 and 2670 cm^–1^ are attributed to aliphatic groups, corresponding to the asymmetric and symmetric stretching of CH_3_^27^. Peaks at 1620 and 1571 cm^–1^ represent C = C stretching in aromatic rings and C=O bonds^28^. The band at 1034 cm^–1^ is assigned to C–O stretching vibrations^29^, while the activated carbon displays hydroxyl group absorption at 1118 cm^–1^, which can be attributed to C–O and phosphate-related (P–O/P–O–C) vibrations and C–H stretching at 671 cm^[–130^, but it may also be consistent with P–O–P/P₂O₅ species^31^. Additionally, the band at 481 cm^–1^ corresponds to the n→π∗ transition of C–O^32^, which may also reflect n→π transitions of oxygen-containing groups, supporting the presence of abundant O- and P-functionalities as further confirmed by XPS.

#### Determination of pHpzc

The point of zero charge (pH_PZC_) was determined using the pH drift method. A series of vials were prepared, each containing 5.0 mg of TAC and 10 mL of a 0.1 M NaCl solution, with initial pH values ranging from 2.0 to 12.0. These mixtures were agitated at 200 rpm at room temperature (25.0 ± 1.0 °C) for 24 h. The change in pH (ΔpH = pH_f_ – pH_i_) was plotted against the initial pH (pH_i_), and the pHpzc was derived from the intersection with the x-axis^33^. Identifying the pHpzc of an adsorbent is essential for selecting an optimal solution pH to enhance the adsorption process. When the pH is below the pHpzc, the adsorbent surface is positively charged, favoring the adsorption of anionic dyes (ARS). Conversely, when the pH exceeds the pHpzc, the adsorbent surface becomes negatively charged, which is more conducive to the adsorption of cationic dyes (MB)^34^. The resultsare illustrated in Fig. 3C. The pHpzc value of the TAC is 4.05.

#### X-ray photoelectron spectroscopy (XPS) analysis of TAC

**Fig. 4:**
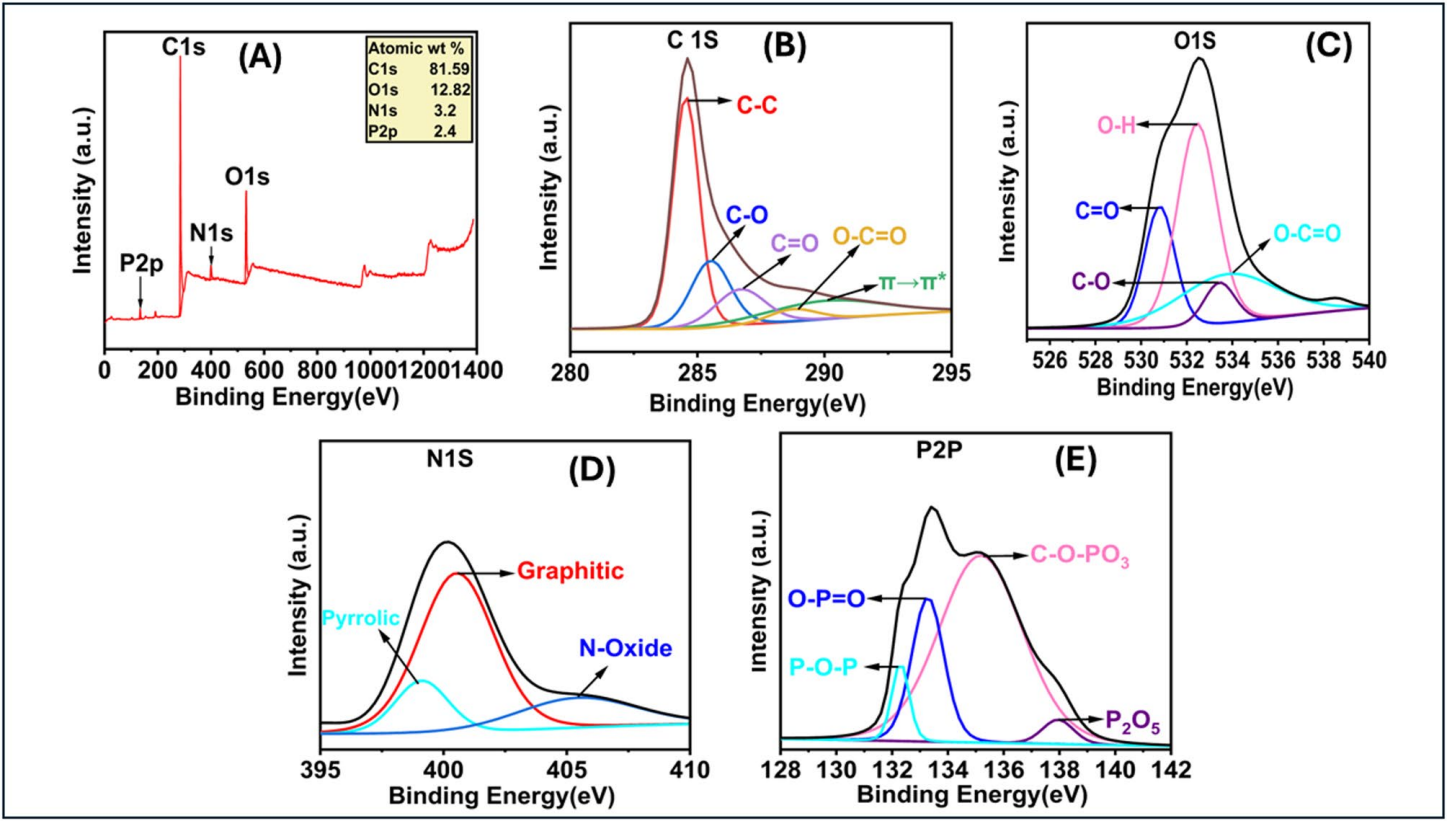
XPS spectra of TAC (A); High resolution XPS spectra of (B) C 1 s, (C) O 1 s, (D) N 1 s, and (E) 2p.

X-ray Photoelectron Spectroscopy (XPS) investigations were conducted to evaluate the synthesized TAC’s surface elemental distribution and chemical structure. The synthetic TAC’s XPS spectrum exhibits peaks at 284.71 eV (C1s), 532.71 eV (O1s), 399.71 eV (N1s), and 133.71 eV (P2p), as depicted in Fig. 4. The C 1 s spectrum was deconvoluted into five distinct peaks, revealing various components: graphitized carbon (284.55 eV), C-O/P (285.53 eV), C = O (286.69 eV), O-C = O (288.75 eV)^35,36^, and π→π* shake-up transitions^37^. The C-O bond signifies the existence of functional groups such as aldehydes, ketones, aliphatic ethers, and carboxylic acids. Moreover, the occurrence of π–π* shake-up transitions indicates a considerable extent of aromatization in the activated carbon. The high-resolution spectra for oxygen display four distinct peaks at 530.82 eV, 532.46 eV, 533.44 eV, and 533.78 eV. These peaks are attributed to C = O/P^38^, C-OH^39^, C-O from alcoholic, phenolic, and etheric groups^40^, and O-C = O in ester and a carboxylic acid^41^, respectively. Consequently, both the FT-IR and XPS spectra validate the existence of carboxylic acid groups functionalized within the TAC adsorbent. The XPS spectra of N1S were deconvoluted into three nitrogen functionalities: pyridinic N (398.2 eV), pyrrolic N (399.12 eV), graphitic N (400.54 eV), and N-oxide (405.44 eV). TAC exhibits a significant presence of N-graphitic (63.5%), succeeded by N-Oxide (19.05%), with a minor proportion of pyrrolic-N (14.99%)^42,43^.The high-resolution P 2p XPS spectra exhibit peaks at 132.3 eV, 133.28 eV, 135.18 eV, and 137.95 eV, which match phosphorus’s binding energies. The peaks correspond to P-O-P, O-P = O^44^, C-O-PO_3_, and PxOy species^45^, respectively.

#### SEM analysis of activated carbons derived from *Phragmites australis* tassels

Activated carbons (ACs) derived from *Phragmites australis* tassels (TPA) are synthesized using a two-step chemical activation process with phosphoric acid (H_3_PO_4_) as the activating agent. This method involves an initial carbonization step followed by activation, providing enhanced control over the development of porosity in the activated carbon^46^. During carbonization, the biomass undergoes structural stabilization and the formation of initial porosity, while the subsequent activation phase further expands and optimizes these pores.

The carbonization process is conducted via pyrolysis under a high-temperature inert environment, facilitating the breakdown of structural barriers and converting micropores into larger mesopores and macropores. This approach results in a material with significantly higher surface area and porosity compared to single-step activation methods. Additionally, the two-step activation process promotes the formation of surface functional groups that enhance adsorption capacities, making these activated carbons highly effective for pollutant removal applications, such as wastewater treatment, where strong adsorbate-adsorbent interactions are critical^47^.

**Fig. 5:**
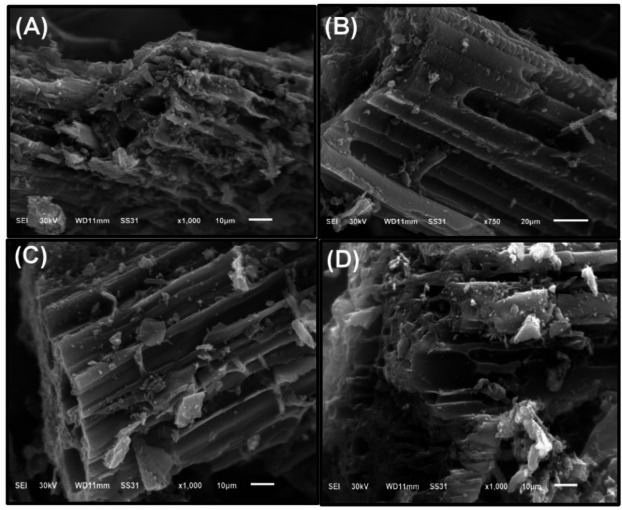
SEM images of activated carbons derived from *Phragmites australis* tassels (A-D).

The SEM images (Fig. 5) reveal that the external surfaces of the activated carbons exhibit numerous irregular cavities, a characteristic attributed to the activation with H_3_PO_4_. The interaction between concentrated phosphoric acid and cellulose leads to depolymerization and the formation of cellulose phosphate esters^48^. During activation, oligomerized H_3_PO_4_ ​ decomposes into P_2_O_5_ and water. The sublimation of P_2_O_5_ creates additional pore structures, while water evaporation further contributes to porosity development. Moreover, the XPS spectra confirm the presence of P–O and P = O bonds with binding energies in the range of 132–137 eV, which can be ascribed to residual phosphate species formed during H₃PO₄ activation. This comprehensive activation process results in activated carbons with enhanced structural and functional properties, optimizing their performance in pollutant adsorption applications^49^.

### Batch adsorption investigations for ARS and MB dyes

#### Effect of pH solution on TAC

**Fig. 6:**
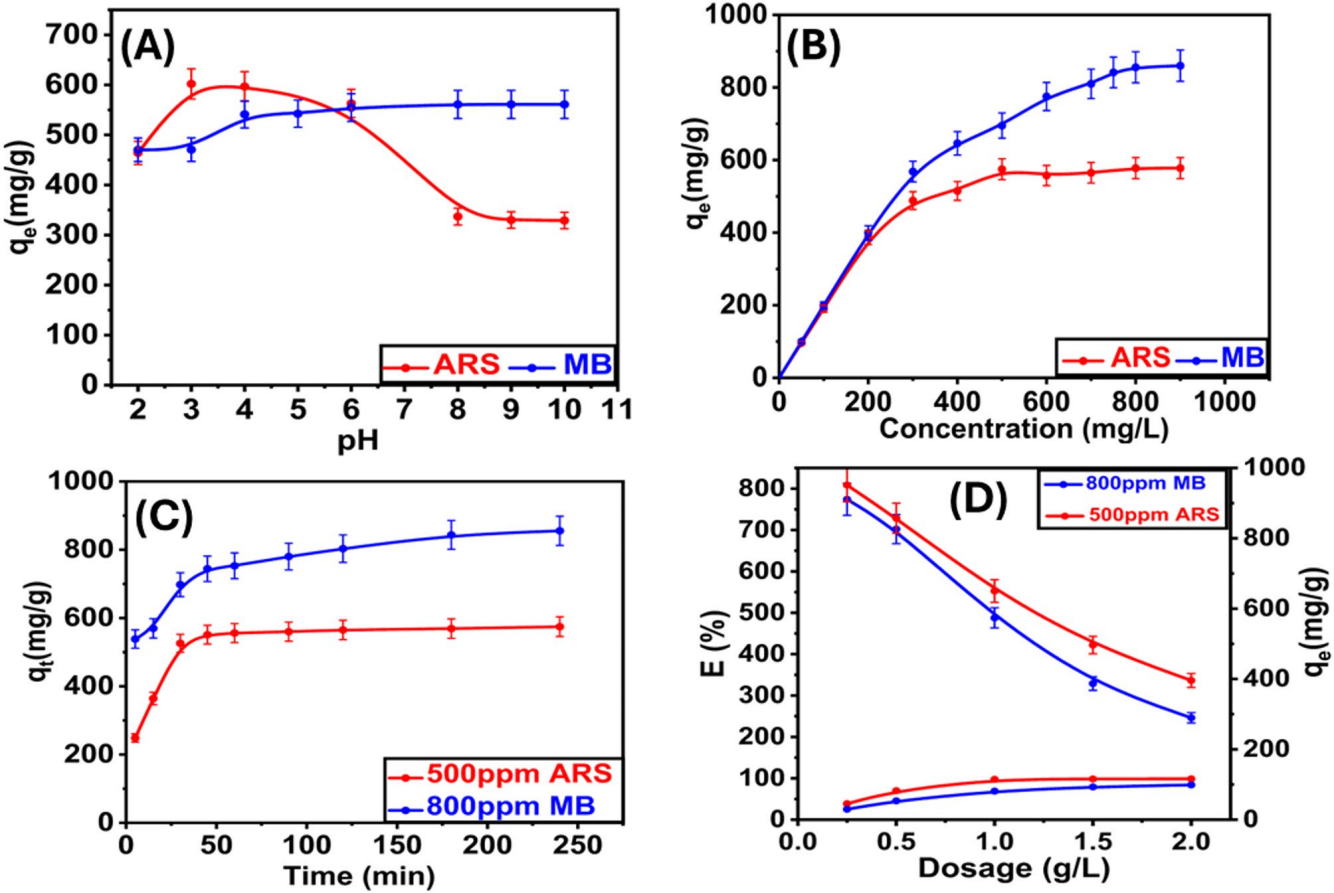
The adsorption efficiency of TAC as a function of (A) pH; (B) concentration, (C) contact time, and (D) Adsorbent dosage.

This investigation focused on the influence of solution pH on the adsorption behavior of dyes utilizing TAC. As illustrated in Fig. 6A, the removal efficiency for ARS was optimal at pH 4, whereas MB exhibited maximum removal at pH 8. pHpzc for TAC was established at 4.05, as indicated in Fig. 2C. When (pH_solution < pHpzc), protonation of the adsorbent occurs. For ARS, this protonation enhances electrostatic interactions between the negatively charged oxygen atoms in the dye’s sulfonic groups and the protonated sites on TAC, thereby promoting increased adsorption under acidic conditions^8^. Conversely, for MB, protonation of carboxyl groups at lower pH levels results in a positively charged surface on TAC, which reduces its negative charge and consequently its affinity for MB molecules. In contrast, when (pH_solution > pHpzc), TAC surfaces become negatively charged due to the deprotonation of carboxyl groups. Under these conditions, electrostatic interactions for ARS are diminished, leading to a reduction in the attractive forces between the adsorbent and the dye. However, this negative charge facilitates electrostatic attraction with positively charged dye ions for MB, thus enhancing removal efficiency^50^.

#### Effect of initial ARS and MB concentration

The influence of the initial concentrations of ARS and MB on removal efficiency was investigated in the range of 50–900 mg/L. This concentration range was carefully selected to cover both environmentally relevant levels commonly reported in textile and dyeing effluents and higher levels that simulate worst-case industrial discharge scenarios. Such a choice ensures that the performance of TAC is evaluated under realistic conditions, while also allowing assessment of its maximum adsorption capacity under more challenging pollutant loads (see Fig. 6B).

The results indicated a significant decline in the removal efficiency for ARS, which decreased from 96.59% to 30.1%. Conversely, the removal efficiency diminished from 99.52% to 47.7% for MB as the initial dye concentrations escalated from 50 to 900 ppm. Additionally, the equilibrium adsorption capacity increased markedly, rising from 96.59 mg/g to 541 mg/g for ARS and from 99.1 mg/g to 860 mg/g for MB. This increase can be attributed to the heightened concentration gradient, which enhances the driving force for adsorption at higher initial concentrations of dyes^51^.

#### Effect of contact time

The influence of contact time on the adsorption capacity of ARS and MB by TAC is depicted in Fig. 6C. The experiments were performed using initial dye concentrations of 500 ppm for ARS and 800 ppm for MB at a controlled temperature of 25 °C. It was observed that the quantity of each dye adsorbed increased with prolonged contact times, reaching equilibrium after 90 min for both ARS and MB. Therefore, a contact time of 90 min was established as the optimal condition for all subsequent experiments. As illustrated in Fig. 6C, the removal efficiency of ARS improved from 24.85% to 58.1%, while the removal percentage for MB increased from 33.64% to 53.09%. Initially, the rate of dye removal was higher due to the greater surface area available on the adsorbent. However, after the initial phase of adsorption, the rate of dye uptake became limited by the transport of dye molecules from the external surface to the internal sites within the adsorbent particles^52^.

#### Effect of adsorbent dosage

The influence of TAC dosage on the adsorption efficacy of ARS and MB is depicted in Fig. 6D. As the mass of TAC increased from 0.25 to 2 g/L for the ARS pollutant, the removal percentage (% R) increased markedly from 38.7% to 99%. The adsorption efficiency for MB varied between 29.72% and 98.97%. The increase is mainly attributable to the improved specific surface area of the TAC, which offers additional adsorption sites for the capture of both ARS and MB. It is essential to recognize that TAC’s adsorption capacity may diminish with increased dosages^53^. Consequently, establishing the appropriate dosage is crucial for industrial applications. Utilizing over 0.005 g (0.5 g/L) of adsorbent may result in superfluous economic expenditure.

#### Adsorption isotherms

Adsorption isotherms are essential instruments for examining the interactions between adsorbents and adsorbates. They offer significant insights into the characteristics of these interactions, and the maximum amount of adsorbate that can be adsorbed. Diverse adsorption isotherm models elucidate the nature of adsorption, the underlying mechanisms, the adsorbent’s affinity, and whether the process is defined by monolayer or multilayer development, in addition to the maximum adsorption capacity. In this context, three models—Langmuir, Freundlich^54^, and Temkin—were utilized to analyze the experimental data (Figure S2). Specifics of these isotherm models are available in Table 1. The experimental data indicate that the adsorption behavior of TAC conforms well to the Langmuir model, exhibiting a good correlation coefficient (R²)^55,56^. The maximum adsorption capacities (q_m_) established by the Langmuir model aligned with the equilibrium adsorption capacities (q_e_).As shown in Table 1, the R_L_ values varied from 0 to 1, indicating that the adsorption processes of ARS and MB onto TAC were advantageous. A reduced R_L_ value indicates a more potent adsorption driving force. This shows that a monolayer chemical adsorption process occurred, signifying the existence of uniform adsorption sites on the adsorbent. Moreover, based on the Temkin constants ($\:{B}_{T}$), It can be deduced that the adsorption mechanism for TAC is classified as exothermic physical adsorption^57^.

The equilibrium data were analyzed using Langmuir, Freundlich, and Temkin isotherm models:

3 $$\:\frac{{C}_{e}}{{q}_{e}}=\frac{1}{{K}_{L}{Q}_{m}}+\frac{{C}_{e}}{{Q}_{m}}$$

4 $$\:\text{ln}{q}_{e}=\text{ln}{K}_{f}+\frac{1}{n}\text{ln}{C}_{e}$$

5 $$\:{q}_{e}=\frac{RT}{{B}_{T}}Ln\left({A}_{T}\right)+\frac{RT}{{B}_{T}}Ln\left({C}_{e}\right)$$

where q_m_ (mg/g) is the maximum adsorption capacity, K​_L_ (L/mg) is the Langmuir constant, K_F​_ ((mg/g) (L/mg)¹/ⁿ) and n are the Freundlich constants, while A_T_​ (L/g) and B (J/mol) are Temkin constants.

**Table 1:** Adsorption isotherm parameters for the removal of MB and ARS by TAC.

Isotherms	Parameter	TAC	
		ARS	MB
Langmuir	$\:{q}_{m}\left({mg.g}^{-1}\right)$	584.795	584.795
	$\:{R}_{L}$	0.0108	0.0128
	$\:{R}^{2}$	0.999	0.994
	$\:{Q}_{exp}(mg.{g}^{-1})$	577.8	860.0
Freundlich	$\:{K}_{F}({mg}^{-1}.L)$	137.590	264.262
	$\:1/n$	0.251	0.202
	$\:{R}^{2}$	0.769	0.798
Temkin	$\:{B}_{T}(J.{mol}^{-1})$	76.96673	65.674
	$\:{A}_{T}(L.{mg}^{-1})$	5.066	6.455
	$\:{R}^{2}$	0.885	0.913

#### Adsorption kinetics

Kinetic models, such as pseudo-first-order (PFO), pseudo-second-order (PSO), and intra-particle diffusion (IPD) models, were utilized to correlate the experimental adsorption data (Figure S3). The correlation coefficient (R²) for the PSO kinetic model, derived from the kinetic parameters calculated in Table 2, is close to one and higher than that of the PFO model. This indicates that the PSO model is more suitable for describing the adsorption of the two types of dyes. The kinetic data suggest that the adsorption of ARS and MB onto TAC occurs through chemisorption. Furthermore, the experimental value for adsorption capacity aligns more closely with the value derived from the PSO model fitting, indicating that the PSO model more accurately characterizes the kinetic process of TAC^58^.

The kinetic data were fitted using pseudo-first-order (PFO), pseudo-second-order (PSO), and intraparticle diffusion (IPD) models:

6 $$\:{\text{l}\text{n}(q}_{e\:\:\:}-{q}_{t})=ln{q}_{e}-{K}_{1}t$$

7 $$\:\frac{t}{{q}_{t}}=\frac{1}{{K}_{2}{{q}_{e}}^{2}}+\frac{1}{{q}_{e}t}$$

8 $$\:{q}_{t}={K}_{diff}*{t}^{1/2}+C$$

Where q_t​_ (mg/g) is the adsorption capacity at time (min), q_e_​ (mg/g) is the adsorption capacity at equilibrium, k_1​_ (1/min) is the pseudo-first-order rate constant, k_2_​ (g/mg·min) is the pseudo-second-order rate constant,$\:{K}_{diff}$ (mg/g·min^0.5^) is the intraparticle diffusion rate constant, and C is the intercept related to the boundary layer effect.

**Table 2:** Adsorption kinetic parameters for the removal of MB and ARS by TAC.

Kinetic model	Parameter		TAC	
			ARS	MB
PFO	$\:{q}_{e,cal}\left({mg.g}^{-1}\right)$		138.299	315.617
	$\:{K}_{1}\:\left({min}^{-1}\right)$		0.021	0.0173
	$\:{R}^{2}$		0.731	0.960
PSO	$\:{q}_{e,cal}\left({mg.g}^{-1}\right)$		588.235	877.193
	$\:{K}_{2}(g/mg.min)$		0.00032	0.00015
	$\:{R}^{2}$		0.999	0.999
IPD	First stage	$\:{K}_{diff,1}\:({mg.g}^{-1}.{min}^{-1/2})$	11.075	9.447
		C_1_$\:\left({mg.g}^{-1}\right)$	194.979	824.689
		$\:{R}^{2}$	0.999	0.969
	Second stage	$\:{K}_{diff,2}$	0.190	0.812
		C_2_	543.286	706.268
		$\:{R}^{2}$	0.873	0.982
	Third stage	$\:{K}_{diff,3}$	0.081	0.436
		C_3_	555.028	755.500
		$\:{R}^{2}$	0.9799	0.822

The analysis of the intraparticle diffusion model (Figure S3 (C)) shows that the plot of q_t_ against t^1/2^ does not create a straight line that intersects at the origin. This observation indicates that IPD is not the only rate-limiting factor in the adsorption process. The adsorption of textile dye contaminants, specifically ARS, MB, and TAC, can be divided into three distinct phases^59^. In the initial phase, ARS/MB molecules migrate to the surface of the adsorbent through a boundary layer and are rapidly adsorbed. A higher value of C_1_ at elevated initial concentrations suggests a thicker boundary layer^60^, which can hinder the rate of diffusion. During the second phase, ARS/MB diffuses into the pores of the activated carbon. This stage is characterized by a slower rate of adsorption as the molecules navigate through the porous structure of the adsorbent. In the final phase, the adsorption process approaches equilibrium^61^. At this stage, both external mass transfer and intraparticle diffusion contribute to the overall rate of adsorption. However, it is important to note that the impact of the intraparticle diffusion stage, indicated by very low values of K_3_, is minimal on the overall kinetics^62^. The constants associated with the intraparticle diffusion model are detailed in Table 2, providing further insight into these dynamics. This comprehensive analysis highlights that while intraparticle diffusion plays a role in adsorption kinetics, it is not solely responsible for controlling the rate; rather, it operates alongside other mechanisms in a complex interplay during the adsorption process.

#### Thermodynamic studies

The thermodynamic factors governing the adsorption of ARS and MB on TAC were investigated by analyzing the adsorption across a temperature spectrum of 298 to 328 K. Figure S4(A) illustrates the influence of temperature on the equilibrium adsorption capacity ($\:{q}_{e}$), demonstrating that the adsorption capacity of ARS rises with elevated temperature. Conversely, for MB, there are minor reductions in ($\:{q}_{e}$) when the temperature increases as depicted in (Figure S4(B)). Thermodynamic parameters, such as Gibbs free energy (ΔG°), enthalpy (ΔH°), and entropy (ΔS°). Figure S4(C) illustrates the linear correlation between the natural logarithm of the distribution coefficient ($\:ln{K}_{d}$) and the inverse temperature (1/T), with the computed findings detailed in the table. The negative Gibbs free energy values for ARS indicate that adsorption happens spontaneously and becomes increasingly favorable with rising temperature (Table 3). Additionally, ΔG values drop with increasing temperature, indicating that the adsorption process becomes more spontaneous at elevated temperatures. Meanwhile, the positive values of ΔS° and ΔH° show that the adsorption process is endothermic. Enthalpy values ranging between 20 and 40 kJ/mol show that the interactions are indicative of electrostatic interactions, or physisorption^63,64^. In the case of MB, it is noteworthy to notice that an increase in temperature leads to a higher ΔG value for its adsorption on TAC, showing that the process becomes less favorable at raised temperatures (Table 3). At lower temperatures, the adsorption of MB shows larger negative ΔG values, implying a stronger adsorption propensity on TAC^60^. The negative ΔH° values show that the adsorption process is exothermic, whereas the negative ΔS° values indicate a more ordered arrangement of molecules on the adsorbent’s surface.

**Table 3:** Thermodynamic parameters for the removal of MB and ARS by TAC.

	T(K)	K_d_	∆G^°^(kJ/mol)	∆H^°^(kJ/mol)	∆S^°^ (J/Kmol)
ARS	298	2.703	−2.463	13.428	53.523
	318	4.196	−3.791		
	328	4.313	−3.986		
MB	298	2.299	−2.062	−20.108	−60.749
	318	1.253	−0.595		
	328	1.125	−0.321		

### Mechanism of adsorption

A comprehensive analysis of the results reveals that a combination of physical and chemical processes governs the adsorption characteristics of ARS and MB on TAC. Fourier Transform Infrared (FTIR) spectroscopy and X-ray Photoelectron Spectroscopy (XPS) were employed to gain deeper insight into the adsorption mechanisms associated with TAC. FTIR analysis was employed to examine the surface properties of TAC, demonstrating a high surface area and the presence of specific functional groups such as hydroxyl, carboxylic, phenol, and ester. The predominant physical mechanism is pore-filling adsorption, facilitated by TAC’s elevated (BET) surface area and its abundant micropores. With an average pore diameter ranging from 1.2 to 3 nm, TAC is particularly effective for adsorbing ARS, which has a molecular size of approximately 1.05 nm × 0.54 nm^65^, as well as MB, which measures around 0.7 nm ×1.7nm^66^. Consequently, minimal pore exclusion effects are observed.

Numerous interactions between dye molecules and activated carbon significantly influence the adsorption mechanism. These interactions encompass electrostatic attraction, π–π interactions, electron donor-acceptor mechanisms, and hydrogen bonding. When the pH of the solution is below (pHpzc) of activated carbon, its surface becomes positively charged. This positively charged surface facilitates electrostatic attraction between tassel-activated carbon and the negatively charged sulfonate groups present in ARS. Conversely, MB, a cationic dye, may experience electrostatic repulsion when interacting with the positively charged TAC surface. At pH levels exceeding the pHpzc, TAC’s surface becomes negatively charged, enhancing electrostatic interactions with the positively charged nitrogen atoms in MB, and promoting its uptake. However, under these conditions, ARS may encounter electrostatic repulsion due to the negative charge of the TAC surface^52,67^. Additionally, the aromatic structures of both ARS and MB facilitate π–π interactions with the π-electrons of the carbon structure^52^. This interaction can further enhance adsorption through charge transfer between the π-electrons of the dyes and those of activated carbon. The combination of these mechanisms—electrostatic attraction, π–π interactions, hydrogen and n–π collectively defines the complex dynamics of dye adsorption onto activated carbon^51,68^. Understanding these interactions is essential for optimizing adsorption processes in wastewater treatment applications involving various dyes^69^.

The adsorption mechanism of ARS and MB onto the prepared tassel-activated carbon (TAC) was elucidated based on FTIR and XPS analyses. For ARS, the FTIR spectra of TAC before and after adsorption (Figure S5) showed clear spectral changes indicating the involvement of several surface functional groups. The pristine TAC exhibited a broad band at ~ 3354 cm^–1^ corresponding to the stretching vibration of –OH groups, peaks at 2922 cm^–1^ and 2247 cm^–1^ related to C–H and C ≡ C stretching, and a band near 1571 cm^–1^ assigned to aromatic C=C vibrations. After ARS adsorption, the –OH peak shifted to 3362–3740 cm^–1^ with decreased intensity^70^, and the C = O/C = C region around 1571 cm^–1^ became broader^71^. New bands appeared near 1172 cm^–1^ and 1067 cm^–1^, confirming the interaction between the sulfonate groups of ARS and the oxygen-containing groups of TAC. These spectral variations indicate that hydrogen bonding, electrostatic attraction, and π–π stacking are the main forces governing ARS adsorption onto TAC^72^.

**Fig. 7:**
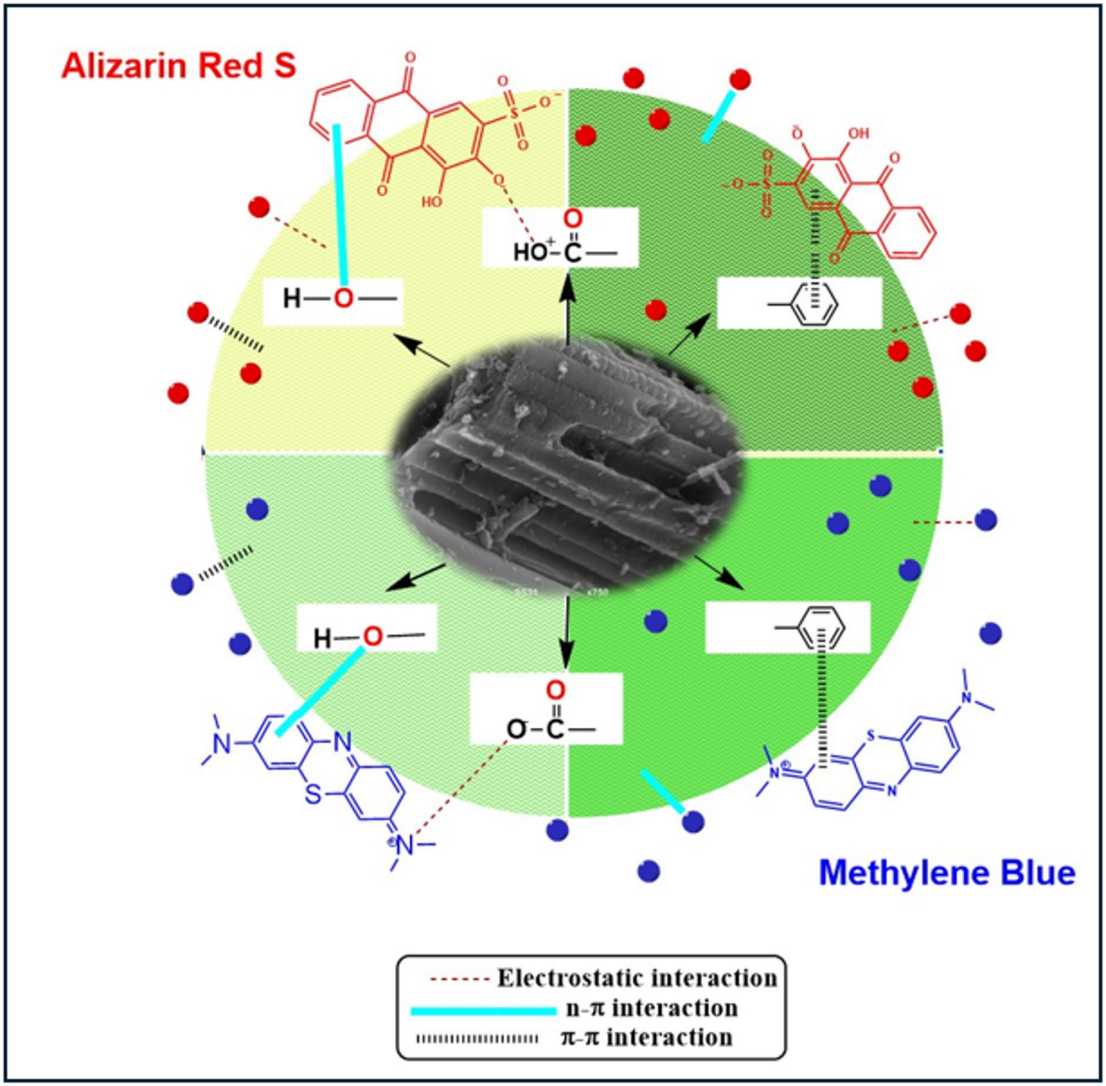
Possible mechanisms for the adsorption of MB and ARS dyes onto TAC.

**Fig. 8:**
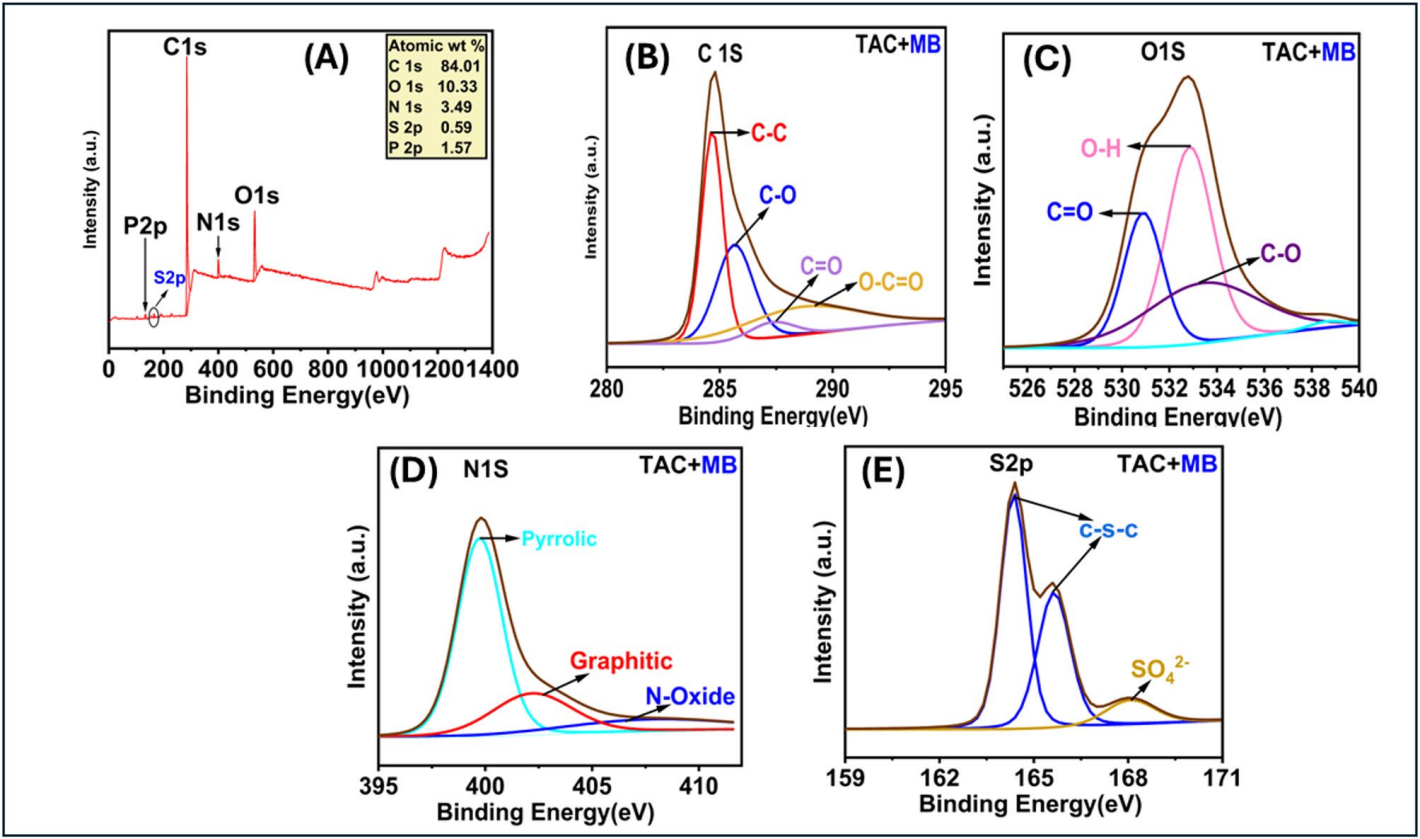
XPS spectra of TAC after MB adsorption (A); High-resolution XPS spectra of (B) C 1 s, (C) O 1 s, (D) N 1 s, and (E) S 2p.

The X-ray photoelectron spectroscopy (XPS) analysis conducted following MB removal identified two significant spectral peaks. The first peak is a doublet observed at 164.36 eV and 165.64 eV, which indicates the presence of carbon (C-S-C) bonds^73^. The second peak is located at 168.07 eV, associated with high-valence sulfur linked to sulfate^74^. These findings confirm the presence of methylene blue on the surface of the activated carbon (Fig. 8A). Moreover, the XPS analysis indicates a substantial reduction in the peak intensity of graphitic nitrogen content, decreasing from 63.5% to 23.53% in the TAC after the adsorption process (Fig. 8A). This decline can be attributed to the Lewis acid-base interaction between graphitic nitrogen on TAC, acting as a Lewis base, and MB, which serves as a Lewis acid, resulting in the gradual depletion of pyridinic nitrogen during adsorption. Conversely, there is a notable increase in the peak intensity of pyrrolic nitrogen from 14.99% to 62.43%, alongside an increase in nitrogen oxide content, highlighting the high adsorption capacity of TAC for MB. Additionally, the aromatic structure of methylene blue facilitates π-π interactions with graphitic nitrogen^75^, further enhancing adsorption efficiency. Furthermore, XPS spectra reveal a reduction in the content of C–OH and O–C = O groups^76^, as indicated by the O1s peak, which corresponds to active oxygen-containing functional groups (Fig. 8C). This finding suggests that these functional groups readily engage in hydrogen bonding with methylene blue (MB) molecules, thereby enhancing the adsorption process of TAC toward MB. Additionally, the peaks observed in the C1s spectra shifted to 284.67, 285.66, 287.33, and 288.33 eV (Fig. 8B). This shift in the spectral positions suggests the presence of electrostatic interactions between the negatively charged oxygen-containing groups on the surface of TAC and methylene blue^77^ (Fig. 7). These conclusions are strongly supported by the experimental results obtained in this study. The FTIR and XPS analyses confirmed the participation of oxygen- and nitrogen-containing functional groups (C–O, O–C = O, graphitic-N, and pyrrolic-N) and revealed new S2p peaks after MB adsorption, consistent with dye–carbon interactions. Moreover, the pH-dependent adsorption behavior further verifies the role of electrostatic attraction in the overall mechanism.

### Application in real water samples

**Fig. 9:**
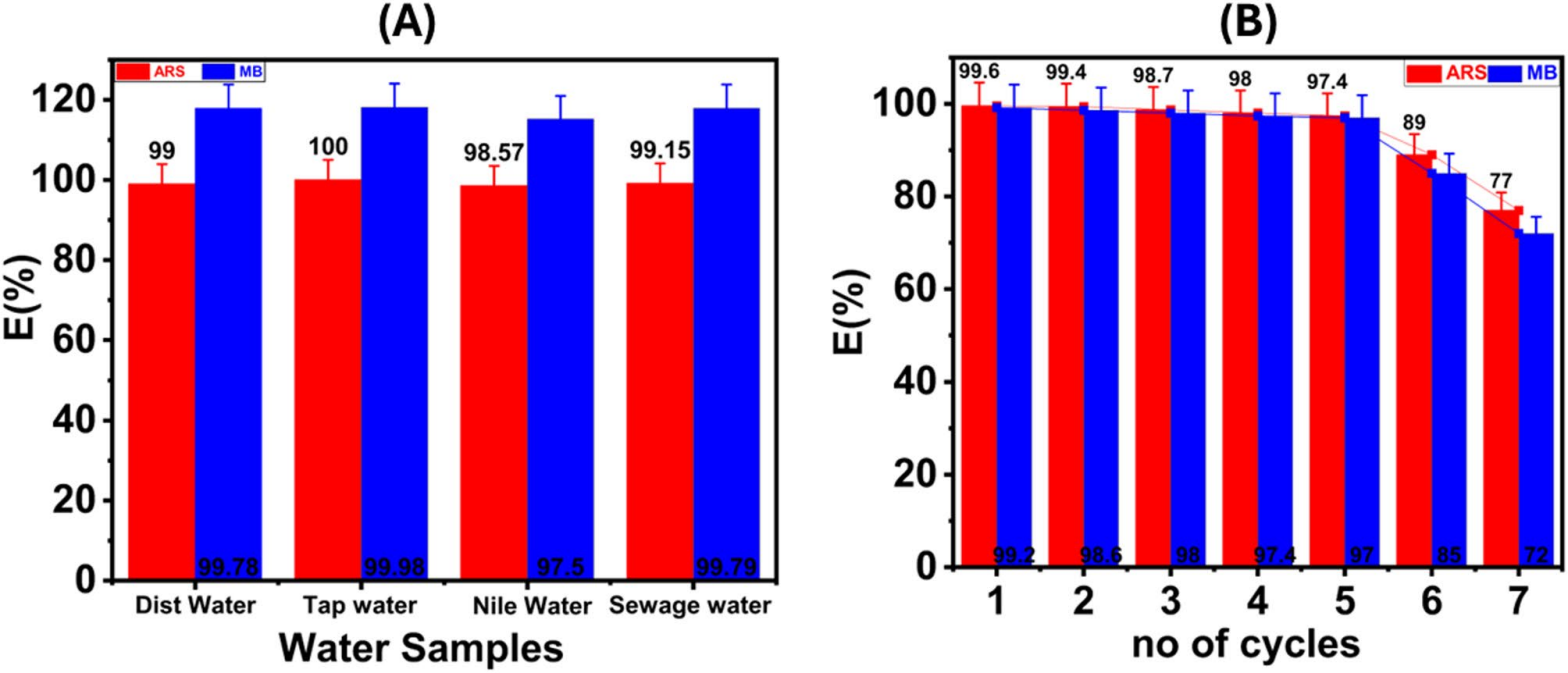
(A) Removal of MB and ARS from real water samples using TAC adsorbent; (B) The reusability of TAC adsorbent for MB and ARS dyes.

In our investigation of the interactions between ARS, MB, and TAC under optimal conditions, we examined a variety of water sources, including tap water, Nile water, and sewage water (Fig. 9A). To facilitate our analysis, we prepared a reference solution containing 100 ppm of ARS and MB. The samples were collected from tap water, a nearby section of the Nile, and an Egyptian sewage treatment facility for laboratory evaluation. Our assessment of TAC’s performance in these real-world water samples yielded notable results. As illustrated in Table S2, TAC demonstrated exceptional dye removal efficiencies, underscoring its practical applicability. TAC has shown considerable efficiency in removing dyes from wastewater. This study highlights the potential of TAC as an effective strategy for dye elimination across diverse water sources in practical applications.

### Desorption and regeneration studies

We assessed the industrial applicability of TAC by investigating its reusability. This study involved seven cycles of adsorption and elution using the optimal sample. After each cycle, we quantified the adsorption capacity to evaluate the viability and reusability of the adsorbent. We conducted desorption experiments on the dye adsorbed by TAC after separating the adsorbent using a magnet to test reusability. We added 50 mg of ARS/MB that had been adsorbed onto TAC to a 100 mL glass vial containing 40 mL of 0.1 M NaOH for ARS and 0.1 M HCl for MB. The mixture was stirred for 2 h at 25 °C to ensure complete desorption. Following this, the suspension was separated by centrifugation. After washing the adsorbents several times with distilled water, we dried the regenerating adsorbent in an oven at 80 °C before using it for further adsorption trials. We examined the efficiency of the adsorbent up to seven cycles at an initial concentration of 100 ppm andan adsorbent dosage of 0.5 g/L over 30 min. In the first five cycles, the adsorbent demonstrated good stability and reusability in removing both anionic and cationic dyes. However, after these cycles, its performance declined rapidly (Fig. 9B**).**

### Cost analysis of tassel-activated carbon preparation

The cost evaluation of tassel-activated carbon (TAC) derived from the tassels of *Phragmites australis* was primarily based on the raw materials and processing chemicals used. Since these tassels are an abundant and invasive agricultural by-product, their feedstock cost can be considered negligible, consistent with previous studies on low-value waste^78^. The main contributors to the production cost of TAC include the activating agent (H₃PO₄), energy consumption during pyrolysis, and post-treatment steps such as washing and drying.A preliminary economic analysis suggests that chemical activation with phosphoric acid, although more expensive than physical activation, yields highly porous carbons with enhanced adsorption capacities, reaching up to 860 mg/g for methylene blue (MB) and 541 mg/g for acid red S (ARS). This high efficiency reduces the amount of adsorbent needed, thereby lowering overall water treatment costs, which aligns with findings regarding other bio-waste-derived carbons^12^. Furthermore, TAC exhibits strong regeneration ability, maintaining over 85% of its removal efficiency after seven cycles. This characteristic enhances its economic viability by reducing replacement costs. Since the precursor can be collected without transportation or purchase fees (through local harvesting of *Phragmites australis*), the production cost of TAC remains relatively low compared to commercial activated carbons, which typically range from 2 to 10 USD per kilogram^79^. Therefore, TAC presents a sustainable and affordable option for dye removal, with financial benefits further amplified by the advantages of waste reuse and invasive species management^80^.

### Comparison with literature

Table 4 presents a comparative overview of the (q_max/exp_) values for ARS and MB adsorption onto TAC adsorbents in relation to other biomass-derived materials reported in previous studies. The results clearly highlight the exceptional efficiency of TAC in removing ARS and MB from aqueous solutions. This superior performance not only demonstrates the novelty of our material but also emphasizes its potential as a low-cost and sustainable alternative for wastewater treatment.

**Table 4:** Comparison of the adsorption performances of TAC with other similar adsorbents for the removal of MB and ARS.

Pollutant	Adsorbent	q_m_ )mg/g)	Surface area(m^2^/g)	pH	Regeneration	References
ARS	MgO/ZnO nanocomposite Fe_3_O_4_/NiO composite PEI@MCNTs NiFe_2_O_4_/PANI ATCDW **TAC**	378.79 222.30 196.08 186.0 87.54 **541.0**	546.8 111.56 127.93 21.94 94.2 **1166.16**	2 3 $\:\le\:$6 4–8.6.6 2 **4**	6 6 4 6 6 **7**	^20^ ^21^ ^81^ ^82^ ^83^ **This study**
MB	ZnO _0.10_/BC _0.90_ BC MC CCMCS-SiO_2_@PAMPS SBC SMK MBC SA-RPB **TAC**	118.8 86.95 285.6 363.67 84.2 370.85 353.4 191.49 **860.0**	286.4 323.04 62 11.46 8.71 6.31 94.2 0.74 **1166.16**	7 7 11 11 11 9 8 - **8**	4 - 4 - **-** **-** 4 - **7**	^84^ ^85^ ^86^ ^87^ ^88^ ^22^ ^89^ ^90^ **This study**

## Conclusion

This study demonstrates that tassel-activated carbon (TAC), synthesized from *Phragmites australis* tassels, is a highly efficient and sustainable adsorbent for removing ARS and MB from wastewater. Its remarkable textural properties, including a surface area of 1166.16 m²/g and a pore volume of 1.5038 cm³/g, together with its hierarchical porosity and abundant functional groups, enabled adsorption capacities that exceeded those of many conventional adsorbents. Optimal performance was achieved at pH 4 for ARS and pH 8 for MB, with adsorption processes well described by the Langmuir isotherm and pseudo-second-order kinetics, confirming monolayer chemisorption as the dominant mechanism. Furthermore, TAC exhibited excellent regeneration and structural stability over multiple cycles, underscoring its potential for cost-effective and long-term application in wastewater treatment. Despite these promising results, certain limitations should be acknowledged. The present experiments were restricted to small-scale batch studies with synthetic dye solutions. Future work will therefore focus on batch scale-up and continuous-flow systems to validate TAC’s feasibility in larger volumes and under real industrial conditions, as well as extending its use to other contaminants. The value-added contribution of this research lies in transforming an abundant and invasive biomass into a high-performance adsorbent, thereby addressing both dye pollution and waste valorization. By advancing a renewable, eco-friendly, and reusable material, this study supports sustainable water purification, fosters circular economy practices, and aligns with the principles of green chemistry. TAC thus emerges as a practical and scalable candidate for mitigating dye-laden wastewater and contributing to cleaner aquatic environments.

## Supplementary Information

Below is the link to the electronic supplementary material.

Supplementary Material 1

## Data Availability

All data generated or analyzed during this study are included in this published article [and its supplementary information files].

## References

[1] Wu, J., Annath, H., Chen, H. & Mangwandi, C. Upcycling tea waste particles into magnetic adsorbent materials for removal of cr (VI) from aqueous solutions. *Particuology* **80**, 115–126 (2023).

[2] Bakry, A. M. et al. Enhanced performance of amine and thiol chemically modified graphene oxide for effective removal of hg (II), Pb (II), and cr (VI) from aqueous solution. *Appl. Water Sci.* **14**, 179 (2024).

[3] Liu, Y. et al. Simultaneous and ultrafast removal of anionic and cationic dyes from aqueous solution by Zr-based MOFs hybridized by attapulgite and adsorption performance research. *Colloids Surf., A*. **680**, 132643 (2024).

[4] Meili, L. et al. Adsorption of methylene blue on agroindustrial wastes: experimental investigation and phenomenological modelling. *Prog. Biophys. Mol. Biol.* **141**, 60–71 (2019). 30055187 10.1016/j.pbiomolbio.2018.07.011

[5] Shelke, B. N., Jopale, M. K. & Kategaonkar, A. H. Exploration of biomass waste as low cost adsorbents for removal of methylene blue dye: A review. *J. Indian Chem. Soc.* **99**, 100530 (2022).

[6] Oladoye, P. O., Ajiboye, T. O., Omotola, E. O. & Oyewola, O. J. Methylene blue dye: toxicity and potential elimination technology from wastewater. *Results Eng.* **16**, 100678 (2022).

[7] Santoso, E. et al. Review on recent advances of carbon based adsorbent for methylene blue removal from waste water. *Mater. Today Chem.* **16**, 100233 (2020).

[8] Pourebrahim, F., Ghaedi, M., Dashtian, K., Heidari, F. & Kheirandish, S. Simultaneous removing of Pb2 + ions and Alizarin red S dye after their complexation by ultrasonic waves coupled adsorption process: spectrophotometry detection and optimization study. *Ultrason. Sonochem.* **35**, 51–60 (2017). 27765487 10.1016/j.ultsonch.2016.09.002

[9] Zhang, S., Shen, X., Li, J. & Zhang, J. Study on degradation of Alizarine reds in simulated dye wastewater by gas-liquid two-phase discharge plasma. *Chem. Eng. Processing-Process Intensif.* **181**, 109114 (2022).

[10] Kurniawan, T. A. et al. Resource recovery from landfill leachate: an experimental investigation and perspectives. *Chemosphere* **274**, 129986 (2021). 33979934 10.1016/j.chemosphere.2021.129986

[11] Mbarki, F., Kesraoui, A., Seffen, M. & Ayrault, P. Kinetic, thermodynamic, and adsorption behavior of cationic and anionic dyes onto corn stigmata: nonlinear and stochastic analyses. *Water Air Soil Pollut.* **229**, 1–17 (2018).

[12] Tigrine, Z. et al. Sustainable activated carbon from agricultural waste: A study on adsorption efficiency for humic acid and Methyl orange dyes. *Sustainability* **16**, 9308 (2024).

[13] Gopal, K. et al. Development of a new efficient and economical magnetic sorbent silicone surfactant-based activated carbon for the removal of chloro-and nitro-group phenolic compounds from contaminated water samples. *RSC Adv.* **9**, 36915–36930 (2019). 35539062 10.1039/c9ra07151b PMC9075134

[14] Weerasuk, B., Chutimasakul, T., Prigyai, N. & Sangtawesin, T. Enhanced dye removal and supercapacitor performance of polyethyleneimine-impregnated activated carbon derived from local Eucalyptus Biochar. *RSC Sustain.* **3**, 904–913 (2025).

[15] Grich, A. et al. Preparation of low-cost activated carbon from doum fiber (Chamaerops humilis) for the removal of methylene blue: optimization process by DOE/FFD design, characterization, and mechanism. *J. Mol. Struct.* **1295**, 136534 (2024).

[16] Andersen, L. H. et al. Can Reed harvest be used as a management strategy for improving invertebrate biomass and diversity? *J. Environ. Manage.* **300**, 113637 (2021). 34521006 10.1016/j.jenvman.2021.113637

[17] Čížková, H., Kučera, T., Poulin, B. & Květ, J. Ecological basis of ecosystem services and management of wetlands dominated by common Reed (Phragmites australis): European perspective. *Diversity* **15**, 629 (2023).

[18] Rezania, S. et al. Phytoremediation potential and control of phragmites australis as a green phytomass: an overview. *Environ. Sci. Pollut. Res.* **26**, 7428–7441 (2019). 10.1007/s11356-019-04300-4 30693445

[19] Moheb, M., El-Wakil, A. M. & Awad, F. S. Highly porous activated carbon derived from the Papaya plant (stems and leaves) for superior adsorption of Alizarin red s and methylene blue dyes from wastewater. *RSC Adv.* **15**, 674–687 (2025). 39781019 10.1039/d4ra07957d PMC11708045

[20] Al-Kadhi, N. S. et al. Facile synthesis of MgO/ZnO nanocomposite for efficient removal of Alizarin red S dye from aqueous media. *Inorg. Chem. Commun.* **162**, 112233 (2024).

[21] Nodehi, R., Shayesteh, H. & Rahbar-Kelishami, A. Fe3O4@ NiO core–shell magnetic nanoparticle for highly efficient removal of Alizarin red S anionic dye. *Int. J. Environ. Sci. Technol.* **19**, 2899–2912 (2022).

[22] Bülbül, A., Delibaş, A. & Coşkun, R. Development and characterization of activated charcoal adsorbent derived from oak for efficient removal of methylene blue: functionality vs surface area. *Biomass Convers. Biorefinery*, 1–16 (2025).

[23] Al-Qarhami, F., Khalifa, M. E., Abdallah, A. & Awad, F. S. Remediation of wastewater containing methylene blue and acid Fuchsin dyes using 2-aminothiazole chemically modified Chitosan. *Int. J. Biol. Macromol.* **303**, 140744 (2025). 39919401 10.1016/j.ijbiomac.2025.140744

[24] Nouioua, A. et al. Production of Biochar from melia Azedarach seeds for the crystal Violet dye removal from water: combining of hydrothermal carbonization and pyrolysis. *Bioengineered* **14**, 290–306 (2023). 37477231 10.1080/21655979.2023.2236843 PMC10364649

[25] Gao, J. et al. Adsorption of methylene blue onto activated carbon produced from tea (Camellia sinensis L.) seed shells: kinetics, equilibrium, and thermodynamics studies. *J. Zhejiang Univ. Sci. B*. **14**, 650–658 (2013). 23825151 10.1631/jzus.B12a0225 PMC3709070

[26] Mortada, W. I. et al. Mesoporous magnetic Biochar derived from common Reed (*Phragmites australis*) for rapid and efficient removal of methylene blue from aqueous media. *Environ. Sci. Pollut. Res.* 1–12 (2024). 10.1007/s11356-024-33860-3 PMC11219389 38866933

[27] He, S. et al. Facile Preparation of N-doped activated carbon produced from rice husk for CO2 capture. *J. Colloid Interface Sci.* **582**, 90–101 (2021). 32814226 10.1016/j.jcis.2020.08.021

[28] Zhao, C. et al. Removal of Tetracycline from water using activated carbon derived from the mixture of phragmites australis and waterworks sludge. *ACS Omega*. **5**, 16045–16052 (2020). 32656426 10.1021/acsomega.0c01524 PMC7346240

[29] O. Prakash, M., Raghavendra, G., Ojha, S. & Panchal, M. Characterization of porous activated carbon prepared from Arhar stalks by single step chemical activation method. *Mater. Today: Proc.* **39**, 1476–1481 (2021).

[30] Guo, Z., Zhang, J. & Liu, H. Ultra-high Rhodamine B adsorption capacities from an aqueous solution by activated carbon derived from phragmites australis doped with organic acid by phosphoric acid activation. *RSC Adv.* **6**, 40818–40827 (2016).

[31] Al Lafi, A. G. & Khuder, A. Removal of cr (VI) from aqueous solutions by activated carbon and its composite with P2W17O61: A spectroscopic study to reveal adsorption mechanism. *Heliyon* **11** (2025). 10.1016/j.heliyon.2025.e41862 PMC11773057 39877604

[32] Bhatti, M. A. et al. Highly active carbon material derived from carica Papaya fruit juice: access to efficient photocatalytic degradation of methylene blue in aqueous solution under the illumination of ultraviolet light. *Catalysts* **13**, 886 (2023).

[33] Lawtae, P. & Tangsathitkulchai, C. The use of high surface area mesoporous-activated carbon from Longan seed biomass for increasing capacity and kinetics of methylene blue adsorption from aqueous solution. *Molecules* **26**, 6521 (2021). 34770928 10.3390/molecules26216521 PMC8587158

[34] Doan, V. D. et al. Cu/Fe3O4@ carboxylate-rich carbon composite: one-pot synthesis, characterization, adsorption and photo-Fenton catalytic activities. *Mater. Res. Bull.* **129**, 110913 (2020).

[35] Higai, D., Huang, Z. & Qian, E. W. Preparation and surface characteristics of phosphoric acid-activated carbon from coconut shell in air. *Environ. Prog. Sustain. Energy*. **40**, e13509 (2021).

[36] Liu, X. et al. Reusable salt-template strategy for synthesis of porous nitrogen-rich carbon boosts H2S selective oxidation. *Green Energy Environ.* (2024).

[37] Araújo, M. P., Soares, O., Fernandes, A. J. S., Pereira, M. F. R. & Freire, C. Tuning the surface chemistry of graphene flakes: new strategies for selective oxidation. *RSC Adv.* **7**, 14290–14301 (2017).

[38] González-Domínguez, J. M., Fernández-González, C. & Alexandre-Franco, M. Gómez-Serrano, V. Surface chemistry of Cherry Stone-Derived activated carbon prepared by H3PO4 activation. *Processes* **12**, 149 (2024).

[39] Sun, C., Luo, Z., Yu, P. & Wang, Q. Comparative study on the performance and mechanism of Adsorption–Oriented Phosphorus–Modified High–Efficiency and durable activated Biochar from fast pyrolysis. *Energies* **16**, 5363 (2023).

[40] Guo, T. et al. Investigation of CO2 adsorption on nitrogen-doped activated carbon based on porous structure and surface acid-base sites. *Case Stud. Therm. Eng.* **53**, 103925 (2024).

[41] Zhang, L. et al. Coconut-based activated carbon fibers for efficient adsorption of various organic dyes. *RSC Adv.* **8**, 42280–42291 (2018). 35558414 10.1039/c8ra08990f PMC9092157

[42] Borges Honorato, A. M., Khalid, M. & Pessan, L. A. Coral-like nitrogen doped carbon derived from polyaniline‐silicon nitride hybrid for highly active oxygen reduction electrocatalysis. *Electrochem. Sci. Adv.* **1**, e2000010 (2021).

[43] Liu, B. et al. Hollow-structured cop nanotubes wrapped by N-doped carbon layer with interfacial charges polarization for efficiently boosting oxygen reduction/evolution reactions. *Chem. Eng. J.* **431**, 133238 (2022).

[44] Szczerska, M. et al. Investigation of the Few-Layer black phosphorus degradation by the photonic measurements. *Adv. Mater. Interfaces*. **10**, 2202289 (2023).

[45] Ruiz-Rosas, R., García-Mateos, F. J., Gutiérrez, M. C., Rodríguez-Mirasol, J. & Cordero, T. About the role of porosity and surface chemistry of phosphorus-containing activated carbons in the removal of micropollutants. *Front. Mater.* **6**, 134 (2019).

[46] Odetoye, T. E., Bakar, M. S. A. & Titiloye, J. O. Pyrolysis and characterization of *Jatropha curcas* shell and seed coat. *Nigerian J. Technol. Dev.* **16**, 71–77 (2019).

[47] Hoang, A. T. et al. Remediation of heavy metal polluted waters using activated carbon from lignocellulosic biomass: an update of recent trends. *Chemosphere* **302**, 134825 (2022). 35526681 10.1016/j.chemosphere.2022.134825

[48] Kokol, V., Božič, M., Vogrinčič, R. & Mathew, A. P. Characterisation and properties of homo-and heterogenously phosphorylated nanocellulose. *Carbohydr. Polym.* **125**, 301–313 (2015). 25857987 10.1016/j.carbpol.2015.02.056

[49] Khamkeaw, A., Asavamongkolkul, T., Perngyai, T., Jongsomjit, B. & Phisalaphong, M. Interconnected micro, meso, and macro porous activated carbon from bacterial nanocellulose for superior adsorption properties and effective catalytic performance. *Molecules* **25**, 4063 (2020). 32899569 10.3390/molecules25184063 PMC7570849

[50] Özdemir, M., Durmuş, Ö., Şahin, Ö. & Saka, C. Removal of Methylene blue, Methyl violet, Rhodamine B, Alizarin red, and bromocresol green dyes from aqueous solutions on activated cotton stalks. *Desalination Water Treat.* **57**, 18038–18048 (2016).

[51] Waly, S. M., El-Wakil, A. M., Waly, M. M., El-Maaty, W. M. A. & Awad, F. S. Enhanced removal of Indigo Carmine dye from aqueous solutions using polyaniline modified partially reduced graphene oxide composite. *Sci. Rep.* **15**, 15555 (2025). 40319116 10.1038/s41598-025-98115-8 PMC12049445

[52] Akram, B. et al. Kinetic and thermodynamic analysis of Alizarin red S biosorption by alhagi maurorum: a sustainable approach for water treatment. *BMC Biotechnol.* **24**, 85 (2024). 39478538 10.1186/s12896-024-00913-x PMC11523905

[53] Zhou, Q. et al. Efficient removal of fluoride from water: adsorption performance of MgO-x adsorbents derived from Mg-MOF-74 and insights from DFT calculations. *Sep. Purif. Technol.* **352**, 128190 (2025).

[54] Mackinder, M. A., Wang, K. & Fan, Q. H. Methylene blue adsorption by plasma Re-Activated carbon. *J. Water Resour. Prot.* **13** (2021).

[55] Deniz, S. Efficient and environmentally friendly removal of Azo textile dye using a low-cost adsorbent: kinetic and reuse studies with application to textile effluent. *Mater. Today Commun.* **35**, 106433 (2023).

[56] Huang, J. et al. High-performance Cellulose-based engineering materials derived from coconut shell. *Compos. Part. B: Eng.* 112785 (2025).

[57] Sampranpiboon, P., Charnkeitkong, P. & Feng, X. Equilibrium isotherm models for adsorption of zinc (II) ion from aqueous solution on pulp waste. *WSEAS Trans. Environ. Dev.* **10**, 35–47 (2014).

[58] Jalbani, N. S. et al. Synthesis of new functionalized Calix [4] Arene modified silica resin for the adsorption of metal ions: Equilibrium, thermodynamic and kinetic modeling studies. *J. Mol. Liq.* **339**, 116741 (2021).

[59] Jiang, L. et al. Contributions of various cd (II) adsorption mechanisms by phragmites australis-activated carbon modified with mannitol. *ACS Omega*. **7**, 10502–10515 (2022). 35382289 10.1021/acsomega.2c00014 PMC8973121

[60] Naderahmadian, A. et al. Cellulose nanofibers decorated with SiO2 nanoparticles: green adsorbents for removal of cationic and anionic dyes; kinetics, isotherms, and thermodynamic studies. *Int. J. Biol. Macromol.* **247**, 125753 (2023). 37429351 10.1016/j.ijbiomac.2023.125753

[61] Ahmad, M. A. et al. Adsorption of methylene blue from aqueous solution by peanut shell based activated carbon. *Mater. Today Proc.* **47**, 1246–1251 (2021).

[62] Wang, Y. et al. Efficient removal of acetochlor pesticide from water using magnetic activated carbon: adsorption performance, mechanism, and regeneration exploration. *Sci. Total Environ.* **778**, 146353 (2021). 33725597 10.1016/j.scitotenv.2021.146353

[63] Bellaj, M. et al. Eco-friendly synthesis of clay-chitosan composite for efficient removal of Alizarin red S dye from wastewater: A comprehensive experimental and theoretical investigation. *Environ. Res.* **247**, 118352 (2024). 38309561 10.1016/j.envres.2024.118352

[64] Sayed, N. S. M., Ahmed, A. S. A., Abdallah, M. H. & Gouda, G. A. ZnO@ activated carbon derived from wood sawdust as adsorbent for removal of Methyl red and Methyl orange from aqueous solutions. *Sci. Rep.* **14**, 5384 (2024). 38443380 10.1038/s41598-024-55158-7 PMC10915167

[65] Li, Z. et al. Heat treatment of calcite to enhance its removal of color dye Alizarin red S. *Crystals* **14**, 450 (2024).

[66] Wang, L. et al. Enhanced removal of methylene blue from water by mesopore-dominant Biochar from kelp: Kinetic, equilibrium and thermodynamic studies. *Colloids Surf., A*. **688**, 133652 (2024).

[67] Nizam, N. U. M., Hanafiah, M. M., Mahmoudi, E., Halim, A. A. & Mohammad, A. W. The removal of anionic and cationic dyes from an aqueous solution using biomass-based activated carbon. *Sci. Rep.* **11**, 1–17 (2021). 33883637 10.1038/s41598-021-88084-z PMC8060261

[68] Tagyan, A. I. et al. Potential application of innovative Aspergillus terreus/sodium alginate composite beads as eco-friendly and sustainable adsorbents for Alizarin red S dye: isotherms and kinetics models. *Microorganisms* **11**, 1135 (2023). 37317108 10.3390/microorganisms11051135 PMC10222648

[69] Al-Qarhami, F., Khalifa, M. E., Abdallah, A. B. & Awad, F. S. Remediation of wastewater containing methylene blue and acid Fuchsin dyes using 2-aminothiazole chemically modified Chitosan. *Int. J. Biol. Macromol.* **303**, 140744 (2025). 39919401 10.1016/j.ijbiomac.2025.140744

[70] Bharath Balji, G. & Senthil Kumar, P. Adsorptive removal of alizarin red S onto sulfuric acid-modified avocado seeds: kinetics, equilibrium, and thermodynamic studies. *Adsorpt. Sci. Technol.* **2022**, 3137870 (2022).

[71] Sani, M. & Ayuba, A. M. Adsorption of Alizarin red S dye from aqueous solution using chemically activated typha grass (T. latifolia): equilibrium, kinetic and thermodynamic studies. *Arab. J. Chem. Environ. Res.* **9**, 150–167 (2022).

[72] Dai, K., Rebi, A., Kiran, S., Li, Z. & Han, R. Adsorption efficiency of Alizarin red and 2, 4-dichlorphenoxyacetic acid from wastewater onto Chitosan and iron oxide-coated peanut husk-activated carbon: Isothermal, kinetic, and antibacterial analyses. *Int. J. Biol. Macromol.* 145504 (2025). 10.1016/j.ijbiomac.2025.145504 40581027

[73] Cheng, S., Wang, T., Chu, L., Li, J. & Zhang, L. Preparation of nitrogen-doped activated carbon used for catalytic oxidation removal of H2S. *Sci. Total Environ.* **915**, 170073 (2024). 38242466 10.1016/j.scitotenv.2024.170073

[74] Alegre, C. et al. Influence of nitrogen and sulfur doping of carbon xerogels on the performance and stability of counter electrodes in dye sensitized solar cells. *Catalysts* **12**, 264 (2022).

[75] Li, Z., Xing, B., Ding, Y., Li, Y. & Wang, S. A high-performance Biochar produced from bamboo pyrolysis with in-situ nitrogen doping and activation for adsorption of phenol and methylene blue. *Chin. J. Chem. Eng.* **28**, 2872–2880 (2020).

[76] Yu, H. et al. Experimental and DFT insights into the adsorption mechanism of methylene blue by alkali-modified corn straw Biochar. *RSC Adv.* **14**, 1854–1865 (2024). 38192323 10.1039/d3ra05964b PMC10773387

[77] Wang, J. et al. On the adsorption characteristics and mechanism of methylene blue by ball mill modified Biochar. *Sci. Rep.* **13**, 21174 (2023). 38040771 10.1038/s41598-023-48373-1 PMC10692330

[78] Lai, J. Y. & Ngu, L. H. The production cost analysis of oil palm waste activated carbon: a pilot-scale evaluation. *Greenh. Gases Sci. Technol.* **10**, 999–1026 (2020).

[79] Abubakar, A. M. et al. Measurement: Energy. *Measurement* **7**, 7 (2025).

[80] Al-Ghurabi, E. H., Boumaza, M. M., Al-Masry, W. & Asif, M. Optimizing the synthesis of nanoporous activated carbon from date-palm waste for enhanced CO2 capture. *Sci. Rep.* **15**, 17132 (2025). 40382356 10.1038/s41598-025-00498-1 PMC12085607

[81] Zhang, Z., Chen, H., Wu, W., Pang, W. & Yan, G. Efficient removal of Alizarin red S from aqueous solution by polyethyleneimine functionalized magnetic carbon nanotubes. *Bioresour. Technol.* **293**, 122100 (2019). 31518817 10.1016/j.biortech.2019.122100

[82] Liang, Y., He, Y. & Zhang, Y. -h. Adsorption property of Alizarin red S by NiFe2O4/polyaniline magnetic composite. *J. Environ. Chem. Eng.* **6**, 416–425 (2018).

[83] El-Rayyes, A. et al. Cow Dung waste as an eco-friendly adsorbent for Alizarin red S dye removal. *Sci. Rep.* **15**, 29641 (2025). 40804108 10.1038/s41598-025-14887-z PMC12350782

[84] Essa, R. A., El-Aal, A., Sedky, M., Zeid, A., Amin, S. & E. F. A. & ZnO NPs-modified Biochar derived from banana peels for adsorptive removal of methylene blue from water. *J. Mol. Struct.* **1321**, 139821 (2025).

[85] Sutar, S. & Jadhav, J. A comparative assessment of the methylene blue dye adsorption capacity of natural Biochar versus chemically altered activated carbons. *Bioresource Technol. Rep.* **25**, 101726 (2024).

[86] Almeida, C. A. P., Debacher, N. A., Downs, A. J., Cottet, L. & Mello, C. A. D. Removal of methylene blue from colored effluents by adsorption on montmorillonite clay. *J. colloïd Interface Sci.* **332**, 46–53 (2009). 19150082 10.1016/j.jcis.2008.12.012

[87] Wang, Y. et al. Covalently crosslinked carboxymethyl Chitosan beads containing SiO2 and ionic polymer for efficient adsorptive removal of methylene blue. *Int. J. Biol. Macromol.* 139441 (2025). 10.1016/j.ijbiomac.2024.139441 39755316

[88] Cai, F. et al. Preparation of nitrogen-doped bagasse-derived Biochar with outstanding methylene blue adsorption performance. *Ind. Crops Prod.* **224**, 120415 (2025).

[89] Mortada, W. I. et al. Mesoporous magnetic Biochar derived from common Reed (Phragmites australis) for rapid and efficient removal of methylene blue from aqueous media. *Environ. Sci. Pollut. Res.* **31**, 42330–42341 (2024). 10.1007/s11356-024-33860-3 PMC11219389 38866933

[90] Nguyet, B. T. M. et al. Enhanced adsorption of methylene blue by chemically modified materials derived from phragmites australis stems. *Acta Chim. Slovenica* **69** (2022). 10.17344/acsi.2022.7567 36196799

