# Supplementary material for: Eco-friendly tassel-derived activated carbon for efficient dye removal in wastewater treatment
Source: Sci Rep. 2025 Dec 2;15:43018. doi: 10.1038/s41598-025-28825-6 (PMC12675781; doi:10.1038/s41598-025-28825-6)
Supplement: Supplementary file 1 — Supplementary Material 1 [file 41598_2025_28825_MOESM1_ESM.doc]

**Innovative Tassel-Activated Carbon for Efficient Dye Removal in Wastewater Treatment**

Mona Moheb^1^, Ahmad M. El-Wakil^1^, Saadia M. Waly^1^ and Fathi S. Awad^1*^

^1^Chemistry Department, Faculty of Science, Mansoura University, Mansoura 35516, Egypt.

S1. Experimental analysis and calibration curves

Calibration curves were constructed for Methylene Blue (λ_max_ = 664 nm) and Alizarin Red S (λ_max_ = 424 nm) using standard solutions within the linear absorbance range. The obtained linear relationships (R² > 0.999) were applied to calculate dye concentrations during adsorption experiments^19,20^ .


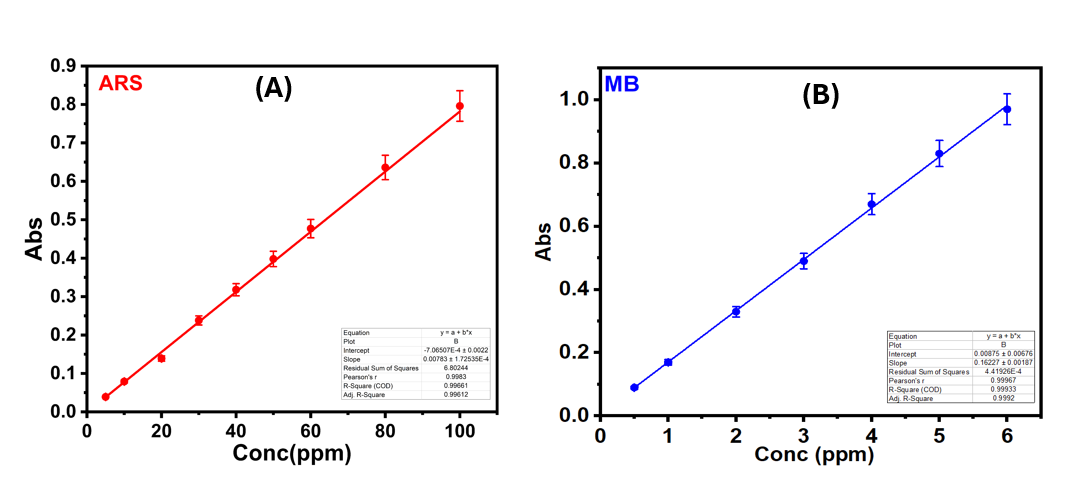


Figure S1. Calibration curve of Alizarin Red S )a) and Methylene Blue (b).

Table S1. Surface characteristics of TAC.

| **Sample** | **TAC** |
| --- | --- |
| BET surface area | 1166.16 m²/g |
| BJH adsorption surface area | 391.627 m²/g |
| Pore volume | 1.13077 cc/g |
| Pore radius | 1.92591 nm |
| DFT adsorption surface area | 952.3615 m²/g |
| Pore Volume | 1.5038 cc/g |


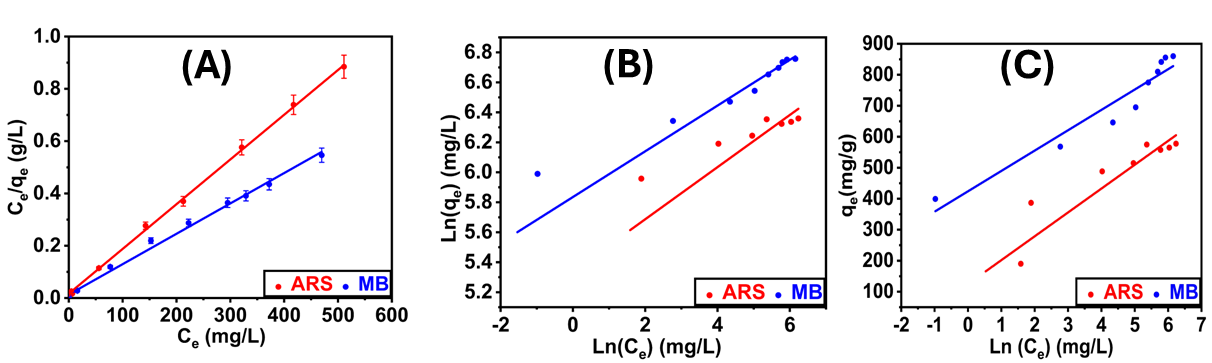


Figure S2. (A) Langmuir isotherm model, (B) Freundlich isotherm model, and (C) Temkin isotherm model.

#
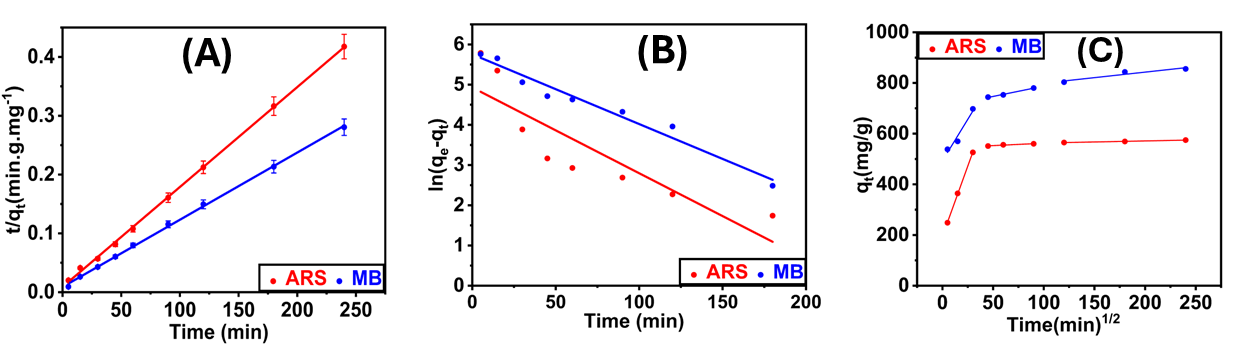


Figure S3. (A) pseudo-second-order (PSO), (B) pseudo-first-order (PFO), and (C ) intra-particle diffusion (IPD) models.


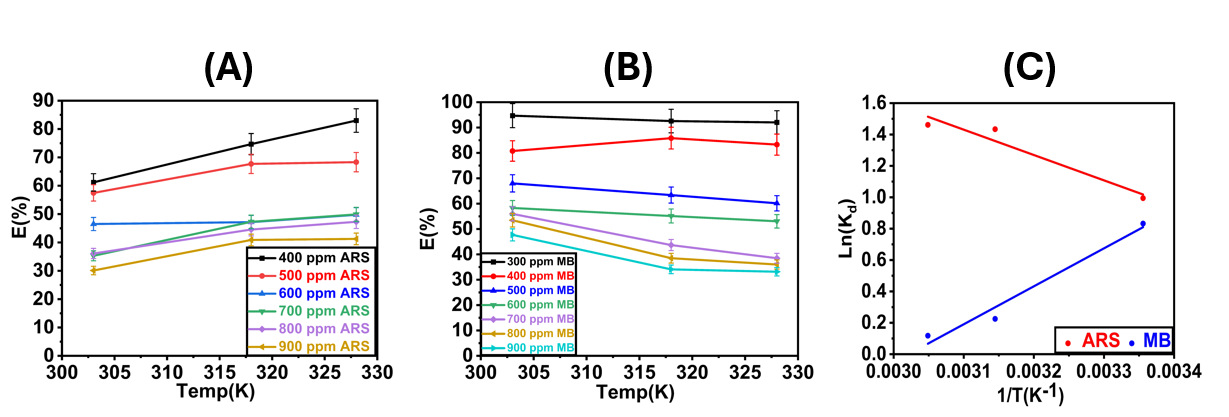


Figure S4. The effect of temperature on the ARS (A) , and MB (B) adsorption onto TAC , and Van^⸴^t Hof plots of ARS and MB (C).


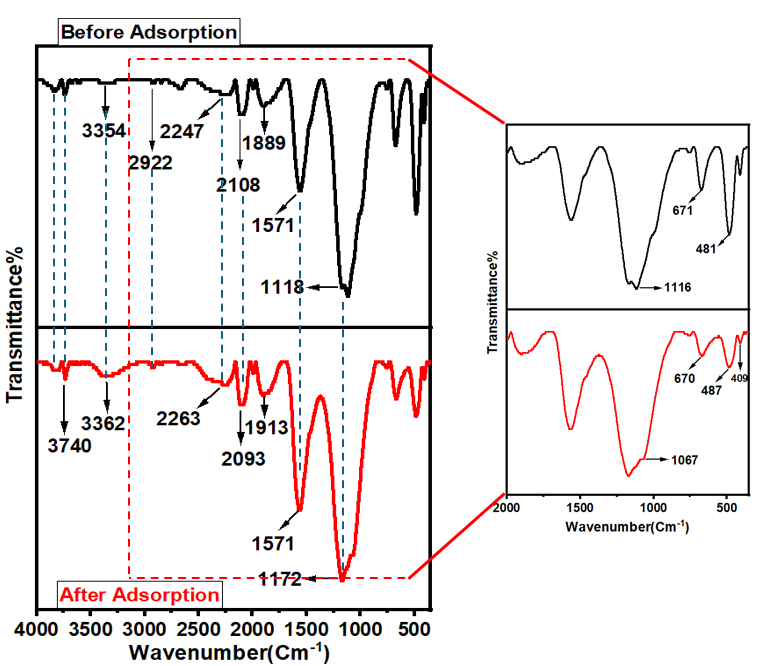


Fig S5: FTIR spectra of TAC before and after ARS adsorption.

Table S2: Removal of MB and ARS from real water samples using TAC adsorbent

| Samples | unspiked | Spiked with 100ppm ARS | ARS | | | Spiked  with 100ppm  MB(DF25) | MB | | |
| --- | --- | --- | --- | --- | --- | --- | --- | --- | --- |
|  |  |  | After removal | % E | RSD% |  | After removal | % E | RSD% |
| Tap water | ND | 0.984 | Zero | 100±0.816 | 0.82 | 0.798 | 0.003 | 99.98±0.531 | 0.53 |
| Nile Water | ND | 0.945 | 0.014 | 98.57±.964 | 0.97 | 0.602 | 0.009 | 99.87±.975 | 0.976 |
| Sewage water | 0.050 | 0.951 | 0.008 | 99.15±0.69 | 0.69 | 0.752 | 0.039 | 99.79±0.535 | 0.536 |
